# Political polarization of news media and influencers on Twitter in the 2016 and 2020 US presidential elections

**DOI:** 10.1038/s41562-023-01550-8

**Published:** 2023-03-13

**Authors:** James Flamino, Alessandro Galeazzi, Stuart Feldman, Michael W. Macy, Brendan Cross, Zhenkun Zhou, Matteo Serafino, Alexandre Bovet, Hernán A. Makse, Boleslaw K. Szymanski

**Affiliations:** 1 Department of Computer Science and Network Science and Technology Center, Rensselaer Polytechnic Institute, Troy, NY USA; 2 University of Brescia, Brescia, Italy; 3 Ca’ Foscari University of Venice, Venice, Italy; 4 Schmidt Futures, New York, NY USA; 5 Departments of Information Science and Sociology, Cornell University, Ithaca, NY USA; 6 School of Statistics, Capital University of Economics and Business, Beijing, China; 7 Levich Institute and Physics Department, City College of New York, New York, NY USA; 8 Department of Mathematics and Digital Society Initiative, University of Zurich, Zurich, Switzerland

**Keywords:** Computer science, Complex networks, Politics and international relations, Complex networks

## Abstract

Social media has been transforming political communication dynamics for over a decade. Here using nearly a billion tweets, we analyse the change in Twitter’s news media landscape between the 2016 and 2020 US presidential elections. Using political bias and fact-checking tools, we measure the volume of politically biased content and the number of users propagating such information. We then identify influencers—users with the greatest ability to spread news in the Twitter network. We observe that the fraction of fake and extremely biased content declined between 2016 and 2020. However, results show increasing echo chamber behaviours and latent ideological polarization across the two elections at the user and influencer levels.

## Abstract (web summary)

Flamino et al. examine changes in the political news media and influencer landscape of Twitter between the 2016 and 2020 US presidential elections.

## Main

A growing number of studies have documented increasing political polarization in the USA that is deeper than at any time since the American Civil War^1–3^. Partisan division over issues has increased among those affiliated with political and news media organizations—elected representatives, party officials and political pundits—alongside an alarming increase in affective polarization among voters^4,5^. This two-level pattern—issue polarization among political elites and affective polarization among voters—invites further research on the diffusion of polarized political information between those in positions of political influence and the larger population.

This diffusion of political information is difficult to track with traditional survey and roll call voting data that lack relational measures. Increasing reliance on social media for political communication is opening unprecedented opportunities to study the diffusion of political information and misinformation^6,7^ over communication networks^8^. Furthermore, the rapid growth of Twitter, Facebook, Reddit and other social media have transformed the communications and information propagation landscape. Alongside traditional broadcast media and face-to-face communication, people now can search for and exchange information with billions of other users in a global network. Recent studies have examined the impact of new technologies, like Twitter and YouTube, on election outcomes^9–18^, including the effects of disinformation^19–25^. Other studies have documented how social media platforms contribute to polarization through the creation of echo chambers^26–35^.

We use a vast amount of social media data collected from Twitter over the 2016 and 2020 US presidential elections enriched with political bias classifications to study diffusion dynamics of political content through news media. In this longitudinal study, we focus on shifts in Twitter’s political landscape caused by changes in the news media content being disseminated. We discovered that, proportionally, the fraction of tweets in the fake news and extremely biased news categories decreased or stayed the same on Twitter.

We also focus on analysing news media influencers, defined as users with the greatest ability to broadly propagate news media information over social media. We analyse changes in their influence, composition and the types of news media they are disseminating between the two elections. We find that the proportion of top influencers affiliated with news media organizations decreased in 2020, while the proportion of those affiliated with political organizations increased. We also quantify and compare the levels of polarization between 2016 and 2020. There are multiple types and levels of polarization established in literature^36–43^, which we discuss in the Supplementary Materials. However, we focus on ‘ideological polarization’^44^ of Twitter users, defined as the level of ideological separation between the political alignments of the content that these users propagate. For the remainder of the article, we use the term ‘polarization’ to refer specifically to ‘ideological polarization’. Our polarization analysis reveals an increase in echo chamber behaviour between 2016 and 2020 resulting from Twitter users’ tendency to be less likely to disseminate information or interact with users on the other side of the political spectrum. This analysis also suggests that new influencers from 2020 are more polarized than the influencers who persisted from the 2016 US presidential election. We believe these results establish a foundation for future work by providing observations on trends and patterns arising in Twitter’s political landscape in news media.

## Results

We note that the initial foundation for this research is established in ref. ^21^, which analysed the news media diffusion dynamics on Twitter during the 2016 US presidential election. We harness part of the data used in that article and follow its relevant methodology to identify and classify influencers in the 2020 US election data. Additionally, following an editorial request added to the reviews of this article, we anonymized all Twitter usernames of personal accounts in both the main manuscript and the Supplementary Materials. Specifically, if the username being presented does not represent an established major news organization that is verified on Twitter, that username is replaced with an alias. This alias consists of two parts: affiliation and year of relevance. A user’s affiliation can be with the media, US politics or personal (see the  News media influencers section for more information on how we define affiliations). The personal affiliation is also split into ‘individual’ and ‘other’ labels, with the former representing no official affiliation with media or politics, and the latter representing a lack of information required to make a distinction. All affiliation labels are shortened to their first five letters in the alias. Year of relevance is determined as being in the top 100 list of influencers for 2016, 2020 or both. See the Twitter retweet networks section for more details on influencers and our influencer identification algorithm. So, a politically affiliated user that was influential only in 2016 will have an alias of ‘Polit_2016’.

### News media on Twitter in 2016 and 2020

We tracked the spread of political news on Twitter in 2016 and 2020 by analysing two datasets containing tweets posted between 1 June and election day (8 November in 2016 and 2 November in 2020). The data were collected continuously using the Twitter search API with the names of the two presidential candidates in each of the presidential elections in 2016 and 2020 as keywords. Using more keywords targeting specific media outlets or hashtags concerning specific news events could miss election-related tweets that did not contain references to the list of outlets or events.

The 2016 dataset contains 171 million tweets sent by 11 million users and was used in refs. ^13,21^ to assess the influence of disinformation on Twitter in 2016. The 2020 dataset contains 702 million tweets sent by 20 million users. Hence, we observe a near doubling of the number of Twitter users involved in spreading political news in 2020 compared with 2016.

At the time we collected our data, the statistical analyses of the raw collected data were limited because the data collection process designed by Twitter itself has been shown to have sampling issues. For instance, the probability of non-responses from API queries is not provided by Twitter, and Twitter has acknowledged that the 100% firehose is not actually a 100% sample, the 10% is not a randomly distributed 10% and the 1% is not a randomly distributed 1%. Thus, standard sampling methods are difficult to apply to the collected Twitter data. However, for the goals of our article, this is our best option as there are no other large-scale, comprehensive datasets available for both the 2016 and 2020 US elections that are readily accessible to us.

The classifications of news media websites presented below and used here, including ‘fake’, ‘extremely biased’, ‘left’ and ‘right’, and especially the boundaries between categories, are a matter of opinion rather than a statement of fact. We use terms ‘left’ and ‘right’ for political leanings that are often referred to as ‘liberal’ and ‘conservative’ on the US political ideology spectrum. The categorizations and labels assigned to the corresponding classes and used here originated in publicly available datasets from fact-checking and bias rating organizations, which are credited below. The classifications of political views and the related conclusions contained in this article should not be interpreted as representing opinions of the authors or their funders.

For each tweet containing a URL link, we extracted the domain name of the URL (for example, www.cnn.com) and classified each link directing to a news media outlet according to this outlet’s political bias. The 2016 and 2020 classifications rely on the website allsides.com (AS), followed by the bias classification from the website mediabiasfactcheck.com (MBFC) for outlets not listed in AS (both accessed on 7 January 2021 for the 2020 classification). We classified URL links for outlets that mostly conform to professional standards of fact-based journalism in five news media categories: right, right leaning, centre, left leaning and left. We also include three additional news media categories to include outlets that tend to disseminate disinformation: extreme bias right, extreme bias left and fake news. Websites in the fake news category have been flagged by fact-checking organizations as spreading fabricated news or conspiracy theories, while websites in the extremely biased category have been flagged for reporting controversial information that distorts facts and may rely on propaganda, decontextualized information or opinions misrepresented as facts. A detailed explanation of the methodologies used by AS and MBFC for rating news outlets and of the differences in classification between 2016 and 2020 is given in the Methods. The full lists of outlets in each category in 2016 and 2020 are given in Supplementary Tables 1 and 2. In the 2016 dataset, 30.7 million tweets, sent by 2.3 million users, contain a URL directed to a media outlet website. The 2020 dataset contained 72.7 million tweets with news links sent by 3.7 million users. This number reveals a drop in the fraction of tweets flowing from users that propagate news media links, from 18% in 2016 to 10% in 2020.

**Fig. 1:**
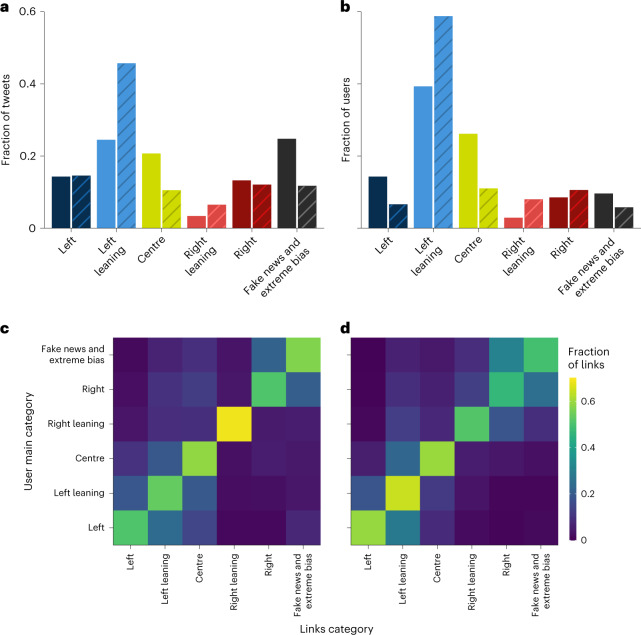
Distribution of news media links in 2016 and 2020 by news media category. **a**,**b**, The fraction of tweets (**a**) and users (**b**) that sent tweets with a URL pointing to a website belonging to one of the categories. Solid coloured bars show fractions for the 2016 election, while striped bars represent the corresponding fractions from the 2020 election. Users are classified as being in the category in which they posted the most links. **c**,**d**, The fractions of links across categories as a function of the users’ main categories, for those users that have at least two links classified, in 2016 (**c**) and 2020 (**d**).

The proportions of tweets and users who sent a tweet in each of the news media categories are shown in Fig. 1a,b along with other statistics about the activity of users in each category. The raw numbers used to generate this figure are shown in Supplementary Table 3. Importantly, they demonstrate that the fraction of tweets in the fake and extremely biased category (representing outlets that were most susceptible to sharing disinformation) decreased from 10% to 6% for fake news and from 13% to 6% for extreme bias right news. The fraction of users who shared those tweets also decreased for extreme bias right news (from 6 to 3%) but not for fake news (which remained at 3%). However, the total number of tweets and users increased over the same period by 411 and 80%, respectively. In short, between 2016 and 2020, the numbers of tweets and users grew at a rate in the range of 80 to 246% for all categories, except the number of users who shared extreme bias right news, which declined by 10%.

The fraction of tweets in the extreme bias left category was only 2% in 2016 and it dropped to a mere 0.05% in 2020. The number of tweets in this category also dropped. The fraction of tweets in the centre category also decreased, from 21 to 10%, but the number of tweets increased dramatically. By contrast, the fraction of left-leaning tweets increased from 24 to 45%, while the fraction of right-leaning tweets increased from 3 to 6%.

The shift away from the centre may indicate the increasing ideological polarization, both among users and media outlets. However, most of the decrease in the fraction of centre media outlets reflects the shift of cnn.com, because it was categorized by AS as centre in 2016 and as left leaning in 2020. CNN accounted for more than twice the number of tweets in 2020 compared to the top outlet of the centre category in that year (thehill.com) (Supplementary Table 2).

Figure 1c,d shows the fraction of URLs for all categories as a function of a user’s modal category for users that posted at least two links in our datasets. The analysis reveals two clusters in 2016 and 2020, one with categories from the right (right leaning, right, fake news and extreme bias) and a second cluster with categories from the centre and left (centre, left leaning and left). These two clusters can be interpreted as two echo chambers in terms of a separation in news consumption. Asymmetrical patterns in Fig. 1c,d above and under the diagonal reveal that users in the right-wing echo chamber also link to an extremely limited number of left-wing media outlets. The users in the left-wing echo chamber link to right-wing media in an even more limited way. This is consistent with asymmetry between left-leaning and right-leaning users in social media observed in previous studies^21,25,35,45^.

To estimate the volume of tweets sent from automated accounts such as bots, we counted the number of tweets sent from unofficial Twitter clients, such as Twitter clients other than the Twitter Web client, Android client, iPhone client or other official clients. Unofficial Twitter clients include those who are using a variety of different applications used to automate all or part of an account activity, such as third-party applications used typically by brands and professionals (for example, SocialFlow or Hootsuite) or bots created with malicious intentions^13^. There is no fast and precise method for bot detection, and the sheer size of our datasets prevented us from using complicated methods, which often use natural language processing, machine learning classifiers and similar techniques^46^. Filtering through unofficial clients provides a simple alternative that meets the baseline requirements for our analyses. Accounts from unofficial clients are only removed during our polarization analysis presented later in the article. For all other analyses, these accounts are kept, as they impact the patterns of information diffusion that we are analysing.

The overall fraction of tweets sent from unofficial clients was 8% in 2016, but this had dropped to 1% in 2020. A similar drop over the same period was observed in the average activity of their users (Supplementary Table 3). This decrease, and the proportional decrease of extremely biased and fake news, could be attributed in part to measures taken by Twitter to limit the virality of disinformation. As mentioned above, the relative volume of tweets linking to disinformation websites dropped by a half in 2020 compared to 2016, and the fraction of users sharing fake news decreased even more substantially (Fig. 1a,b and Supplementary Table 3).

**Fig. 2:**
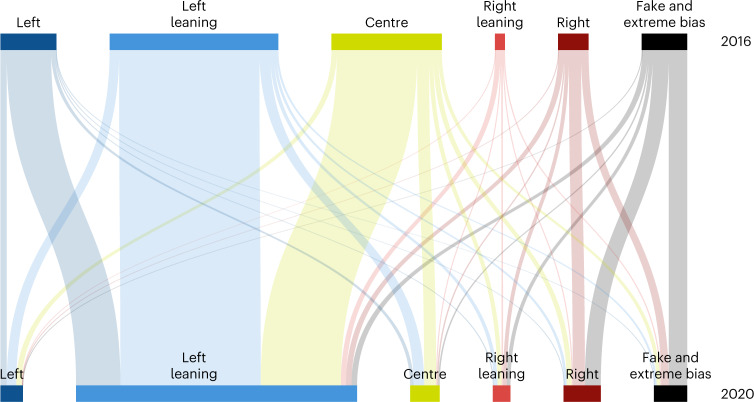
Shifts of users across news media categories from 2016 to 2020. The relative size of each category in 2016 corresponds to the ratio of the numbers of unique users in this category to the left category (Fig. 1). The shifts among categories over time are proportional to the fraction of users that were classified in 2016 and in 2020 in the two involved categories. Overall, 14% of the users present in 2020 persisted from the 2016 dataset.

To understand how users shifted between categories from 2016 to 2020, we track users that were active during both election years (14% of the users present in 2020) and we classified each of them into the category in which they posted the most tweets in each year. Figure 2 shows the resulting shifts. The two largest shifts are among users in the centre and left news category in 2016 that AS rating shifted to the left-leaning category in 2020. This made the left-leaning category the largest in 2020, by shifting the three most widely shared news outlets: *The* *New York Times*, *Washington Post* and *CNN* (Supplementary Table 2). We also observe a large fraction of users in the fake and extremely biased news category in 2016 that moved to the right news category in 2020. However, these user shifts also reflect the change in the classification of media outlets from 2016 to 2020. We infer the ideological position of Twitter users without relying on the news outlet classification (see the subsection Polarization among Twitter users) and show that the resulting positions are highly correlated with the user’s positions computed using the news categories in which they posted.

### Twitter retweet networks

To capture the dynamics of information diffusion, we reconstruct retweet networks corresponding to each news media category. We add a link (a directed edge) going from node *v* to node *u* in the news network when user *u* retweets the tweets of user *v* ≠ *u* containing a URL linking to a website belonging to one of the news media categories. Only one such link is created regardless of the number of tweets retweeted by *u*. With our convention, the direction of the link represents the direction of the influence propagation between Twitter users.

A 2015 study by Metaxas et al.^47^ found that ‘retweeting indicates not only interest in a message, but also trust in the message and the originator, and agreement with the message contents’. Although the retweeting does not explicitly represent support of the retweeted content (since a user who almost always retweets CNN might occasionally retweet Fox News), in a retweet the user cannot remark about reasons for propagating this content to others, while the alternatives of quoting or replying do allow users to remark and so are much more suitable for non-supporting forwarding of news. Accordingly, we assume that most users agree with and are influenced by the information they are propagating through retweets.

Within a network, the in-degree of a node is the number of links that point inward to the node and the out-degree is the number of links that originate at a node and point outward to other nodes. A retweet originates at the node that posted the original tweet, not at the node that posts the retweet (indicating the flow of influence in the direction of the retweeter). Thus, for our retweet networks, the in-degree of a user is equal to the number of users they retweeted at least once and their out-degree is the number of users who have retweeted them at least once. The higher a node’s out-degree, the greater its local influence. The characteristics of the retweet networks are shown in Supplementary Table 4.

We then use an algorithm to find the best spreaders of news media information within each network, that is, the influencers of the corresponding news media category. An alternative of finding the ‘most influential users overall’ through extracting influencers from the retweet networks of all users would result in a list of influencers dominated by left-leaning and centre-biased influencers while other news media bias categories would be underrepresented (see Supplementary Fig. 1, which shows the top overall influencers and their political alignments for both 2016 and 2020). This imbalance would understate the impact of these influencers on polarization between the two election years. Hence, we extract the top influencers from the retweet networks of each news media category to present an accurate representation of critical influencers from the different news media categories. As mentioned earlier, our work builds on and uses some of the results of the 2016 US election from ref. ^21^, which identifies influencers using the Collective Influence (CI) approach^48^. To ensure consistency of results, we too use CI to find influencers in the 2020 data.

### News media influencers

The CI heuristic identifies and ranks influencers in 2016 and 2020 datasets and assigns to each influencer a value CI_out_ that represents the strength of influence it exerts. The influencers identified from these networks only pertain to Twitter accounts who disseminate content by providing links to external sources. We compare the rankings of the influencers extracted from the 2020 network with the rankings of the influencers previously extracted by CI from the 2016 network^21^. A selection of 87 influencers (limited to officially recognized major news organizations that are verified on Twitter as per our disclaimer at the beginning of this section) and their rankings with their corresponding news media categories are shown in Supplementary Table 5.

For the remainder of the article, we use the top influencers extracted from the fake, extreme bias right, right, right-leaning, centre, left-leaning and left news media categories. However, we do not include any influencers from the extreme bias left news media category. According to Supplementary Table 4, this category is sparse and disconnected, with very few users compared to the users’ populations in the other networks. Our goal is to extract influencers that are highly relevant to the dissemination of information on Twitter across the different news media categories. However, we find that the influencers extracted from the extreme bias left category have an extremely low standing in the Twitter community compared to influencers extracted from other categories. For example, the 25th most influential user of the extreme bias left category has about 100 followers, while the 25th most influential user of the left category has over one million. Hence, keeping the extreme bias left category exaggerates the importance of these influencers and diminishes the importance of influencers in other categories. Consequently, we exclude the extreme bias left category from the analyses that follow.

Analysis of our lists of the top influencers in 2016 reveals that traditional news influencers were mostly journalists with verified Twitter accounts linked to traditional news media outlets. By contrast, fake and extremely biased news also contains influencers whose accounts are unverified or deleted, with deceptive profiles and much shorter lifespans on Twitter than traditional media influencers (see Supplementary Figs. 2 and 3 and supporting data in Supplementary Table 6 for details on the proportional shift of users to and from inactivity between election years). However, some of these influencers, despite their unknown, non-public nature, still played an important role in the diffusion of disinformation and information on Twitter^21^. There has been a substantial increase in deleted influencer accounts spreading fake news, from two in the top 25 in 2016 to eight in 2020. Also, the extreme bias right news, which in 2020 consisted primarily of verified influencers, grew from 15 in the top 25 in 2016 to 23 in 2020. We also found that among the top 100 influencers from each news media category in 2020, there was a 29% retention rate of influencers persisting from 2016. Furthermore, for the top 25 influencers from each of these categories, we find the retention rate to be 36%. Meanwhile, as noted earlier, the rate of retention between 2016 and 2020 for the average 2016 user was 14%. The increase in retention rate between the average user and the top 25 influencers is 157%, indicating that the more influential a user, the higher their retention rate.

Using a manual labelling process (see Methods for details), we label the top 25 influencers of each news media category in 2016 and 2020 as affiliated with media or political organizations, or unaffiliated, to observe the makeup of influencer types for these labels. Here, we define an influencer’s ‘affiliation’ with media or politics as their primary job, or other direct connection from which they received periodic financial support. Or, if the influencer is an organizational entity, this classification indicates that this is a legally recognized company. Subsequently, an affiliation indicates if the influencer is either a professional or a legal company outside Twitter.

An influencer affiliated with a media organization could be a media company or official media outlet, or an established writer, reporter or paid consultant. An influencer affiliated with a political party could be a politician, a political campaign platform or an affiliate of the platform, or someone who officially represents an aspect of US politics. We also split the unaffiliated label into two subclassifications: independent and ‘other’. An independent influencer is not officially affiliated with any media or political platforms. The ‘other’ label represents influencers whose accounts have no description or context that could be used to identify them. We generalized these affiliation labels to capture a variation of affiliations to media and politics. It also prevents overcategorization of influencers or the creation of categorization exceptions.

**Fig. 3:**
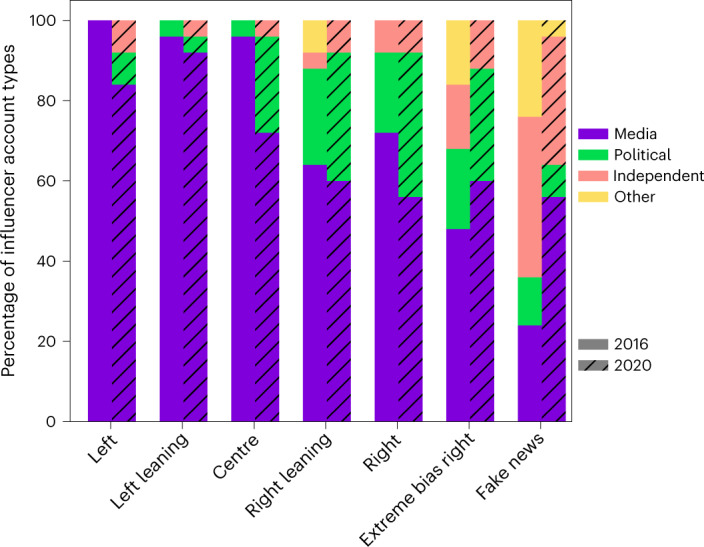
Reshuffling distribution of the top 25 influencer types from 2016 to 2020, by news media category. Influencers are classified as affiliated with a media organization, political organization, independent or other (for example, unidentified).

The fractions of influencers within these affiliation labels are shown in Fig. 3. The results reveal that unaffiliated influencers are more common in the fake and extreme bias news media categories, while affiliated influencers are more common in the other news categories. A similar trend is evident in the fractions of verified and unverified influencers found in these categories, as fake and extreme bias news categories contain fewer verified influencers. In addition, media-affiliated influencers have a greater presence in the left, left-leaning and centre news categories compared with their counterparts.

Interestingly, the number of media-affiliated influencers within most of the news media categories decreased from 2016 to 2020. The exceptions are the extreme bias right and fake news categories, in which the number of media-affiliated influencers increased. Also, the extreme bias right category had increased numbers of politically affiliated influencers. This indicates a shift in polarization of influencers affiliated with right-biased political and media organizations towards the extreme bias right and fake news, as well as the emergence of news media-affiliated influencers in these categories. We discuss these changes in polarization in more detail below.

**Fig. 4:**
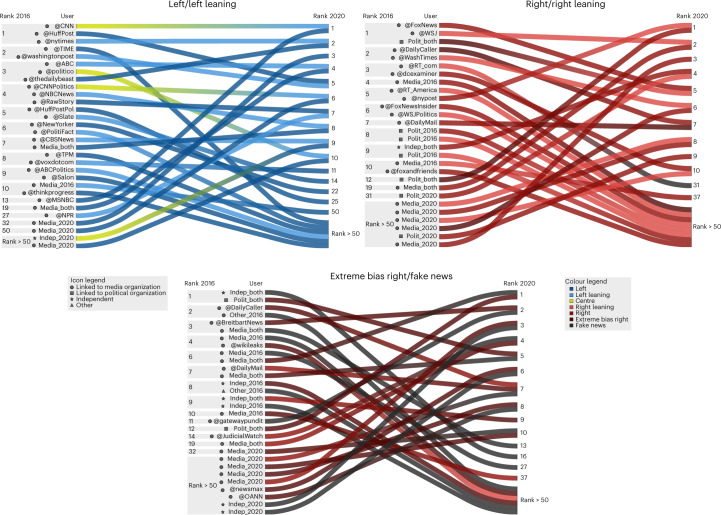
Change in influencers’ rankings from 2016 to 2020. Influencers ranked in the top 10 in at least one news media category in 2016 or 2020 are shown. The 2016 rankings are displayed to the left of the username or alias, with 2020 rankings listed on the right. For each user only one shift is shown. Its colour changes from the user’s highest ranked news media category in 2016 to that in 2020. Each panel shows the change over time between two news media categories.

In addition to changes in affiliations from 2016 to 2020, we observe a substantial reshuffle of the ranking of influencers. Figure 4 shows the change in rankings of the top 10 influencers in left and left-leaning, right and right-leaning, and extreme bias right and fake news categories. The ranking reshuffle in the centre news category is shown in Supplementary Fig. 4.

The comparison reveals several interesting changes between 2016 and 2020. First, we see that highly influential users rise from obscurity. Across all categories, a set of previously unranked or very low-ranked users break into the top 10 rankings. Considering all unique users in the top 25 influential users (from all categories of news media), 58% came from outside the top 100 influential users in 2016. However, most of these newly influential users are related in some way to media or political organizations, while 28% of these new influencers are independent.

Observing the change in rankings by news media category, we see that right and right-leaning, and extreme bias right and fake news categories have a substantally higher fraction of the top 10 influencers who were previously outside the top 50, compared with the change in rankings among the groups in left and left-leaning news categories. All categories show a large number of influencers falling out of the top 50 from 2016 to 2020, and in the case of the left news influencers, we see their former positions filled by users who were much less influential in 2016. The influencers with extreme bias right and fake news affiliations show the most volatility with regards to retaining the top 10 influencer positions, with many top 10 influencers in 2016 ranked below 50 in 2020 (or even banned from Twitter).

The change of classification of some news media outlets is also reflected in the category shifts of their Twitter accounts. In particular, the first- and third-highest ranked influencers in the centre category (@CNN and @politico) in 2016 shifted to left leaning. Such shifts of large and influential media influencers from news categories indicate the increased content polarization on Twitter. A shift of media-affiliated influencers from the right to the extreme bias right is also visible (for example, @DailyMail and @JudicialWatch), as is the emergence of new media-affiliated influencers in these categories (for example, @newsmax and @OANN). In contrast with the shift to the extremes among large media influencers, the centre rankings remained mostly consistent between 2016 and 2020 (Supplementary Fig. 4). Some new users rose from low ranks to fill in the gaps, including the winner of the 2020 US presidential election, but only one user dropped out of the top 50 entirely, and the remaining shifts are internal to these top-ranked users.

### Polarization among Twitter users

The evolution of influencers from different news media categories (Figs. 1 and 2) suggests an increased polarization in the relations among influencers between 2016 and 2020. Here we broaden the scope of polarization analysis to the Twitter users who are consuming and retweeting the influencers’ content. For the 2016 and 2020 data, we consider a set of the top 100 influencers from each news media category. To avoid polarization changes caused by the varying composition of the set of influencers, we filter these sets to contain only influencers that were present in both the 2016 and 2020 CI rankings. For 2016 and 2020, the final set sizes are 505 and 548 influencers, respectively. For this analysis we use all the retweets in our datasets, not only those containing a link to a news outlet, but remove the retweets sent from unofficial Twitter clients.

With influencers as nodes, we create two fully connected similarity networks derived from the 2016 and 2020 Twitter networks, respectively. The weight of an edge between any two influencers in these networks represents the similarity between the retweeters that propagate the content of these influencers (see Methods for more details). Any edges with a weight of 0 are removed. A distribution of the similarity values for both networks, as well as their degree distributions, are shown in Supplementary Fig. 5a,c. In both similarity networks, a community detection algorithm found two communities. One contained influencers affiliated with news media in the centre, left-leaning and left news categories, while the other contained those affiliated with news media in the right-leaning, right and fake news categories. This indicates that influencers separate their user bases according to the content they generate.

**Fig. 5:**
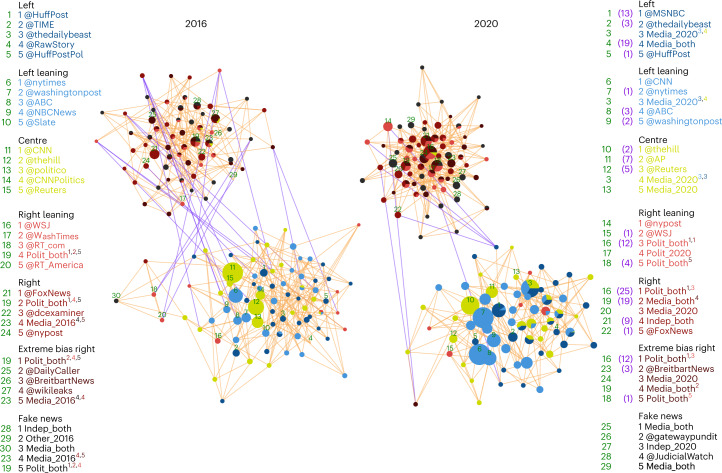
Similarity networks for nodes among the top 25 influencers from each news media category for the two election years. Node size is proportional to its degree in their respective network. Node colour indicates which news media category the node spreads. Nodes that spread information from more than one category are represented as pie charts, where the size of each slice is proportional to their CI score within that respective news media category. An edge between each pair of influencers is weighted by the similarity between the retweeters of those influencers. Both networks are visualized using a force-directed node layout, with the strength of the force defined by the weights of the edges. Since these are complete networks, they are sparsified for visualization purposes, with each node only having up to their five strongest edges visible. Each of the two networks has 405 visible edges in total. Visible intercommunity edges are coloured purple, while intracommunity edges are orange. The distribution of the similarity values, and the degree distributions, for the visible edges of these two networks are shown in Supplementary Fig. 5b,d. Text to the side of each of the two networks shows the top five users of each news media category. A green number to the left of each user corresponds to a labelled node in the network, showing the location of that top influencer. The purple numbers in the 2020 tables indicate the user’s 2016 rank in that category. Users ranked in the top 25 for multiple news media categories have coloured superscripts, indicating the rank and media classification of their other top five positions.

Figure 5 illustrates this separation, showing subsampled similarity networks of the 25 most influential nodes for each news media category for 2016 (left panel) and 2020 (right panel). Using force-directed network layouts driven by the weighted similarity edges, we visualize for both years the two formed communities, one consisting of the right-biased and fake news influencers and the other the left-biased influencers. These communities form an echo chamber motif like the one seen in Fig. 1c,d for the analysis of the fractions of URLs in all news media categories.

Visually, Fig. 5 suggests a loss of intercommunity connections and increased density of intracommunity links from 2016 to 2020. We probe if these changes are reflected in the communities arising in the two main similarity networks containing the nodes from the influencer sets with the top 100 influencers of each news media category. We found that the trends persist, with community separation in the 2020 network increasing compared to the separation of communities in the 2016 network, as measured by modularity and the normalized cut between communities (see Methods for details).

The modularity for the 2020 network was 0.465 with a 95% confidence interval (CI95) of (0.454, 0.475), versus 0.401 with a CI95 of (0.392, 0.409) in 2016, indicating more closely knit communities in 2020, with stronger in-community ties and weaker between-community ties. This trend agrees with the changes of the average normalized cut, which decreased from 0.285 with a CI95 of (0.232, 0.339) in 2016 to 0.052 with a CI95 of (0.046, 0.058) in 2020. Both results show a much stronger separation of the two clusters in the later election and suggest a fundamental shift in retweeting behaviour. Between 2016 and 2020, users became even more likely to disseminate content from influencers with similar biases and less likely to spread content from influencers with opposing biases, effectively reducing cross-bias encounters and discourse. In addition, we also computed the above metrics on networks generated from user quote similarity to confirm that retweets are the strongest form of endorsement of influencer content (Supplementary Table 7). We also report the modularity and normalized cut for the subsampled networks of Fig. 5 in Supplementary Table 8, which reinforces the trend observed in the results above.

To further quantify and compare the changes in user behaviour and, subsequently, in user polarization, we infer the ideology of Twitter users based on the ideological alignment of political actors whom these users follow^29,49^. The bipartite network of followers is then projected on a one-dimensional scale using correspondence analysis^50,51^, which applies singular value decomposition of the adjacency matrix, standardized to account for the differences in popularity and activity of the influencers and their followers (see Methods for details). Two users are close on the resulting latent ideology scale if they follow similar influencers. This method has been shown to produce ideological estimates of the members of the US Congress that highly correlated with ideological estimates based on roll call voting similarity such as DW-NOMINATE^49^.

For 2016 and 2020, the data for the analysis consists of the top 100 influencers of each news media category, as used in the previous polarization analysis, and the sets of users that retweeted at least three different influencers (considering all tweets in our datasets, not only the ones with URLs). As discussed earlier, we interpret retweeting as an endorsement of the content being retweeted. Twitter offers other types of interactions, allowing users to comment on the content, such as quote tweets and replies. The ratio of quotes to retweets of users to influencers was very stable and small (<5%) in 2016 and 2020, for users on both the left and right sides of the latent ideology (Supplementary Table 9A), which motivated our focus on retweets to infer the ideology of users. The ratio of quotes to retweets from users of one side of the ideology spectrum to influencers of the other side increased from 2016 to 2020, indicating an increased usage of quotes to comment on tweets from influencers of the opposite side. However, the overall usage of quotes over retweets remained small (Supplementary Table 9B). We extract the coordinates of each user on the first dimension of the results of the correspondence analysis applied to the weighted network of retweets between the users and the influencers (see Methods for the details and robustness checks that we performed). Finally, for 2016 and 2020, the coordinates of all users are standardized to a mean of zero and a standard deviation of one. Two users are close together on the latent ideology scale if they tend to retweet similar influencers. The influencers’ latent ideological positions are then computed as the median of their retweeters’ positions.

**Fig. 6:**
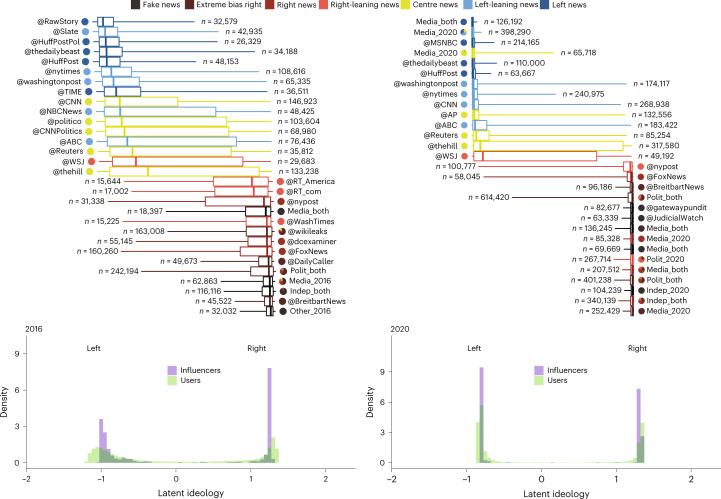
Latent ideology scale of influencers and their retweeters in 2016 (left) and 2020 (right). Top, the latent ideology of the top five influencers of each category is shown as a box plot representing the distribution of the ideology of the users who retweeted them. Bottom, the distributions for the users are shown in green and the distributions for the top 100 influencers of each news media category (computed as the median of the ideology of their retweeters) are displayed in purple. Box plots indicate the median and the 25th and 75th percentiles of the distributions with whiskers indicating the 5th and 95th percentiles. The sample size used for the computation of each box plot is reported to their side. Pie charts next to the influencers’ names represent the news categories to which they belong (weighted by their respective CI ranks in each category).

Figure 6 shows the result of this analysis. The distribution of ideology positions of the users and of the influencers, displayed in green and purple, respectively, shows that polarization increased between 2016 and 2020. This is confirmed by a Hartigans’ dip test (HDT) for unimodality, which measures multimodality in a sample by the maximum difference, over all sample points, between the empirical distribution function and the unimodal distribution function that minimizes that maximum difference^52^. For the user distribution, the test statistic is *D* = 0.11074 with a CI95 of (0.11038, 0.1112) in 2016. In 2020, we have *D* = 0.14751 with a CI95 of (0.1471, 0.1477). For the influencer distribution, the test statistics are *D* = 0.18328 with a CI95 of (0.1672, 0.195) in 2016 and *D* = 0.23251 with a CI95 of (0.206, 0.238) in 2020. All tests reject the null hypothesis of a unimodal distribution with *P* < 2.2 × 10^−16^ and the 95% confidence intervals are computed from 1,000 bootstrap samples using the bias-corrected and accelerated method. Increasing values of the test statistic indicates distributions that increasingly deviate from a unimodal distribution, corroborating the growing division found in the similarity networks.

To resolve whether the increase in polarization is caused by the arrival of new users and influencers in 2020, we repeat the analysis including (1) only users (shown in Supplementary Fig. 6), (2) only influencers (Supplementary Fig. 7) and (3) both users and influencers (Supplementary Fig. 8) that were active during both elections. In all three cases we observe an increase of the HDT statistics (see Supplementary Fig. 9 and Supplementary Table 10) that means that the change in behaviour of the users in selecting whom to send their retweets contributes to the polarization increase. The largest increase in HDT for the user distribution arises when all users from years 2016 and 2020 and only influencers that were present during both years are considered (+0.08). This setting also corresponds to the smallest increase of the dip test of the influencer distribution (+0.01, within CI95), suggesting that the new influencers of 2020 have more polarized ideologies than the influencers who continued from 2016 to 2020. Thus, we conclude that the increased polarization of the users is caused, to a substantial extent, by the arrival and departure of users between elections (Supplementary Fig. 9 and Supplementary Table 10).

Figure 6 reveals a clear increase in polarization of the users and influencers in 2020 compared to 2016 and an alignment of their latent ideologies in two distinct groups, mirroring the news media classification groupings seen in Fig. 5 and Supplementary Fig. 6. This echo chamber behaviour for users became more concentrated in 2020, with two clearly opposite poles that had fewer influencers having a user base bridging opposite ideologies.

These results also independently confirm the shift of news outlets and influencers from the centre to the right and left observed using the news media classifications by external sources. Indeed, we find an extremely high correlation (above 0.90 for 2016 and 2020) between the users’ latent ideology position and their left- or right-leaning distribution computed using the news media categories in which they posted (see Methods for details). This high correlation indicates that the shift in bias observed at the level of the media outlets is also present at the level of the users’ retweeting pattern and serves as an independent validation of the media outlet classification.

## Discussion

We collected and analysed Twitter data during two important US presidential elections in 2016 and 2020 to document changes in Twitter’s political news media landscape and measure the resulting polarization induced by social media influencers and their audiences. Our analyses focus on the identification of influencers and their political alignments. We developed a comparative framework that led to the discovery of important changes in the ideological composition and organizational affiliation of influencers and the level of political polarization among influencers and their audiences. Our descriptive account will be useful for readers and researchers concerned about the power of even a few social media influencers to shape and polarize the news media landscape on Twitter. These results are also an important first step towards the discovery of mechanisms behind the trends, patterns and behaviours we report.

Between 2016 and 2020, the number of influencers affiliated with media organizations declined by 10%, replaced mostly by influencers affiliated with centre and right-leaning political organizations. This change in the news media landscape on Twitter indicates a shift in the relative influence of journalists and political organizations. Among professional media influencers, there was a shift away from independent journalism and towards extreme bias right and fake news.

Importantly, while the number of tweets and users propagating fake and extremely biased news declined between 2016 and 2020, polarization increased across the political spectrum. We found an increase in the division of influencers and users into opposing echo chambers from the first election to the the second. We confirmed this observation by analysing latent ideologies of influencers and users, discovering corresponding increases in their polarization as well. This analysis also suggests that new influencers from 2020 have more polarized ideologies than the influencers who persisted from 2016.

We hope that our results and observations will encourage future studies. One promising direction is to use Natural Language Processing to distinguish between positively and negatively quoted tweets and to determine their topic. Another direction is to extend user classification to refine the categorization of organizational and individual Twitter accounts, and broaden the hierarchy of the users’ affiliations. The measures of polarization can also be refined to enable deeper analysis of the patterns of behaviours that affect polarization and to expand the application of influencer analysis to the spread of political news on other social media.

## Methods

This research was reviewed and classified as exempt by the City University of New York (CUNY) Integrated Institutional Review Boards (IRB), as the research involved the collection of existing data from sources that are publicly available. This decision is shown in IRB file no. 2022-0429 (approved on 8 July 2022) and IRB file no. 2017-0625 (approved on 12 June 2017). These files are available at https://osf.io/e395q/ link.

### News media URL classification

The website www.allsides.com (AS) rates media bias using a combination of several methods such as blind surveys, editorial review, third-party analysis (for example, academic research), independent review and community feedback (see www.allsides.com/media-bias/media-bias-rating-methods for a detailed explanation of the methodology). The website mediabiasfactcheck.com (MBFC) scores media bias by evaluating wording, sourcing and story choices as well as political endorsement (see mediabiasfactcheck.com/methodology). MBFC is maintained by a small independent team of researchers and journalists, offering the largest set of biased and inaccurate news sources among five fact-checking datasets^53^. They are used for labelling bias and veracity of news sources^54–56^. However, neither AS nor MBFC are considered perfect assessments of bias, but no major media assessment platform is considered objectively unbiased from all ideological perspectives. According to the University of Michigan library (an endorser of AS), ‘there is no one exact methodology to measure and rate the partisan bias of news sources’^57^, and as such, we consider AS and MBFC our best options for this research.

To be consistent with the results from 2016^21^, we discard as insignificant outlets that accumulate less than 1% of the cumulative number of tweets of the more popular outlets in each category. Removing uniformly insignificant outlets from all categories also ensures that the tweet volume in each category is independent of the number of outlets classified in this category by AS and MBFC. The full lists of outlets in each category in 2016 and 2020 are given in Supplementary Tables 1 and 2. AS and MBFC updated their bias classification for several outlets between 2016 and 2020, changing the classification used in our analyses as well. For example, CNN Web News was classified in the centre category in 2016 by AS and then in the left-leaning category in 2020, reflecting a bias shift occurring during this time (www.allsides.com/blog/yes-cnns-media-bias-has-shifted-left).

In ref. ^21^, the fake news and extreme bias categories were based on the classification of a team of media experts (available at github.com/alexbovet/opensources) and was cross-checked using the factual reporting scores from MBFC. As the classification source from 2016 was not updated in 2020, we use the list of outlets classified as ‘questionable sources’ from MBFC as a reference for 2020. MBFC describes a questionable source as one ‘that exhibits one or more of the following: extreme bias, consistent promotion of propaganda/conspiracies, poor or no sourcing to credible information, a complete lack of transparency and/or is fake news’. MBFC rates the factual reporting of each source on a scale from 0 (very high) to 10 (very low) based on their history of reporting factually and backing up claims with well-sourced evidence. Outlets with a level of ‘low’ (score of 7 to 9) or ‘very low’ (score of 10) are classified in the fake news category while outlets with a ‘mixed’ level (score of 5 or 6) are classified in the extremely biased category. No outlets in the disinformation categories have a level higher than ‘mixed’. A ‘low’ or ‘very low’ factual reporting level on MBFC corresponds to sources that rarely or almost never use credible sources and ‘need to be checked for intentional fake news, conspiracy, and propaganda’. A ‘mixed’ level is assigned to sources that ‘do not always use proper sourcing or source to other biased/mixed factual sources’. We also verify that all outlets in the extremely biased category have a ‘bias’ reported on MBFC of ‘right’, ‘extreme right’, ‘left’ or ‘extreme left’.

We identify the following in our datasets (giving the top website host name as an example in parenthesis): for the fake news category, 16 website host names in 2016 (top: thegatewaypundit.com) and 20 website host names in 2020 (top: thegatewaypundit.com); for the extreme bias right category, 17 website host names in 2016 (top: breitbart.com) and 10 website host names in 2020 (top: breitbart.com); for the extreme bias left category, 7 website host names in 2016 (top: dailynewsbin.com) and 7 website host names in 2020 (top: occupydemocrats.com); for the left news category, 18 website host names in 2016 (top: huffingtonpost.com) and 18 website host names in 2020 (top: rawstory.com); for the left-leaning news category, 19 website host names in 2016 (top: nytimes.com) and 19 website host names in 2020 (top: nytimes.com); for the centre news category, 13 website host names in 2016 (top: cnn.com) and 13 website host names in 2020 (top: thehill.com); for the right-leaning news category, 7 website host names in 2016 (top: wsj.com) and 13 website host names in 2020 (top: nypost.com); and for the right news category, 20 website host names in 2016 (top: foxnews.com) and 19 website host names in 2020 (top: foxnews.com). The full lists of outlets in each category in 2016 and 2020 are given in Supplementary Tables 1 and 2.

### Influencer identification algorithms

Here, we search for influencers using the Collective Influence (CI) approach^48^, which includes an algorithm that finds a minimal set of nodes that can cause a global cascade in the network operating under the Linear Threshold Model^58^. This task is nondeterministic polynomial-time complete, so the algorithm is prohibitively slow in practice. Thus, we use a computationally efficient CI heuristic that yields an approximate solution. However, the influencer sets used here limit coverage to less than 80% of the global information cascades in our retweet networks. In this range of network cascades, many heuristics, including CI and the popular method PageRank^59^, select similar sets of influencers. As an example, Supplementary Fig. 10 shows that CI and PageRank yield highly correlated lists of influencers, demonstrating that our results do not depend on a choice of heuristic.

### Influencer type classification

For each of the years 2016 and 2020, we manually classified the top 25 influencers in each news media category as either (1) affiliated to a media organization, (2) a political organization, or unaffiliated (classified either as (3) an independent user or as (4) an unidentified ‘other’ user). The manual labelling procedure was as follows: eight of the authors were randomly assigned a subset of the top 25 influencers in these category lists to independently classify to one of the categories (1)–(4), such that three different authors examined each subset. Each author was shown the account name of the influencer along with descriptions, posts and all available non-Twitter information such as their Wikipedia entry. Each influencer was then assigned their category based on the majority vote of the three independent classifications.

### Similarity network analysis

We create for each influencer *i* a vector **S**^*i*^ of size *U*, which stands for the number of users in each election dataset. An index *u* denotes a specific user. The vector element ${{\mathbf{s}}}_{u}^{i}$ defines the number of times user *u* has retweeted influencer *i*. Then, we create the adjacency matrix *W* of size *I* × *I*, where *I* is the number of influencers in each election year dataset. For the 2016 dataset, *I* = 505 while 2020 dataset has *I* = 548. The weight of each edge, and therefore value of each matrix element ${w}_{{i}_{1},{i}_{2}}$, is set to the cosine similarity between vectors ${{{\mathbf{S}}}^{{i}_{1}}}$ and ${{{\mathbf{S}}}^{{i}_{2}}}$. It follows that the higher the cosine similarity of a pair of their user, the more similar are fractions of retweets from influencers *i*_1_, *i*_2_ for this pair. To account for the different number of influencers in two compared election datasets, we extract equally sized random subsets from the two similarity matrices to create the similarity networks used in the following analyses. Each of these networks is limited to a size of 200 nodes. This randomized subset selection process is repeated 100 times. For each pair of similarity networks created in this process, we detect communities in this pair similarity network using the Louvain algorithm^60^. For all 100 pairs of the similarity networks for both election years, we found two communities. Using the accounts of influencers in each community, we found that for both election years one community contains influencers primarily associated with fake and right-biased news categories, while the other contains influencers from centre and left-biased news categories. This split coincides with an underlying division among the Twitter user bases in the content they propagate.

We quantify the severity of this split using two measures of separation between communities. First is modularity that computes the sum of difference between the fraction of edges within each community and the fraction expected within this community in a random network with the same number of nodes and edges. This metric has a range of [−0.5, 1.0]^61^. A positive value indicates the presence of communities separated from each other. The closer the modularity is to 1.0, the stronger communities are separated. The modularity for the 2016 network was 0.401 with a CI95 of (0.392, 0.409). For the 2020 network, it was 0.465 with a CI95 of (0.454, 0.475), in agreement with other methods.

The second measure uses the normalized cut, which is the sum of the weights of every edge that links a pair of communities divided by the sum of the weights of all edges. The result has a range of [0, 1], and the smaller the value, the stronger the separation among communities. The normalized cut for the 2016 network was 0.285 with a CI95 of (0.232, 0.339). However, it decreased to 0.052 with a CI95 of (0.046, 0.058) in the 2020 network.

### Latent ideology estimation

Our latent ideology estimation follows the methods developed in refs. ^29,49^, which rely on retweet interactions instead of following relations. Accordingly, we use correspondence analysis^50^ to infer ideological positions of Twitter users.

The adjacency matrix, *A*, of the retweet network between the influencers and their retweeters is the matrix with element *a*_*ij*_ equal to the number of times user *i* retweeted influencer *j*. We only select tweets that have been sent from the official Twitter client to limit the presence of bots and professional accounts, and we also remove users that show a low interest in the US elections by removing users that retweeted less than three different influencers. For the 2016 data, the matrix *A* has 751,311 rows corresponding to distinct users, 593 columns corresponding to influencers and a total number of retweets equalling 39,385,772. For the 2020 data, the matrix *A* has 2,034,970 rows corresponding to distinct users, 591 columns corresponding to influencers and a total number of retweets equalling 153,463,788.

The correspondence analysis method is executed in the following steps^51^. The matrix of standardized residuals of the adjacency matrix is computed as ${{{{S}}}}={{{{{D}}}}}_{r}^{-1/2}({{{{P}}}}-{{{\bf{r}}}}{{{\bf{c}}}}){{{{{D}}}}}_{c}^{-1/2}$, where ${{{{P}}}}={{{{A}}}}{({\sum }_{ij}{a}_{ij})}^{-1}$ is the adjacency matrix normalized by the total number of retweets. Let **1** denotes a vector of 1’s, **r = 1***P* is the vector of row sums, **c** = **1**^*T*^*P* is the vector of column sums, *D*_*r*_ = diag(**r**) and *D*_*c*_ = diag(**c**). Using the standardized residuals allows the inference to account for the variation of popularity and activity of the influencers and the users, respectively^29^. Then, a singular value decomposition is computed such that *S* = *UD*_*α*_*V*^*T*^ with *UU*^*T*^ = *VV*^*T*^ = *I* and *D*_*α*_ being a diagonal matrix with the singular values on its diagonal. The positions of the users are given by the standard row coordinates: ${{{{X}}}}={{{{{D}}}}}_{r}^{-1/2}{{{{U}}}}$, where we only consider the first dimension, corresponding to the largest singular value. Finally, the ideological positions of the users are found by standardizing the row coordinates to have a mean of zero and a standard deviation of one. The ideological position of the influencers is given by the median of the weighted positions of their retweeters.

To test robustness of this method, we construct three variants of *A* by (1) removing entries with unit weight to discard relations showing a weak ideological alignment; (2) considering the logarithm of the number of retweets as weight for influencer for a sublinear relation between the number of retweets and the strength of ideology alignment; and (3) selecting a random subsample of the 2020 retweet data of the same size as the 2016 retweet data to avoid potential effects of size difference of the two datasets. All robustness tests match the results of our initial method with correlation coefficients between the user position distributions in the robustness tests and in the initial configuration at above 0.995. We also compare the users’ latent ideology distribution with the users’ average leaning distribution and find a correlation above 0.90 for 2016 and 2020. The average leaning of users is computed for all users that have at least three tweets classified in at least one news media category, and is estimated as the weighted average of the news media category positions as the fraction of the distance from the centre, which is assigned bias 0. Each step to the left decreases the bias by 1/3, while each step to the right increases the bias by 1/3, resulting in fractions 4/3 assigned to fake news bias 4/3, 1 to extreme bias right, 2/3 to right bias , 1/3 to right leaning bias, −1/3 to left leaning bias, and −2/3 to left bias.

### Reporting summary

Further information on research design is available in the Nature Portfolio Reporting Summary linked to this article.

## Supplementary information

Supplementary Information Supplementary text, figs. 1–10 and tables 1–10.

Reporting Summary

## Data Availability

The Twitter data is available at https://osf.io/j6pks/ (2016 data) and at https://osf.io/e395q/ (2020 data).

## Code availability

The code used to reproduce the 2016 results are deposited at https://osf.io/e4tvh/, https://github.com/makselab/twitter_opinion_mining and https://github.com/makselab/information_diffusion. The code used to reproduce the 2020 results are at https://osf.io/dbzm2/.

## References

[1] Brady, D. W. & Han, H. C. in *Red and Blue Nation: Characteristics and Causes of America’s Polarized Politics* (eds Nivola, P. S. & Brady, D. W.) **1**(3), 119–141 (Brookings Institute Press, Washington D.C., 2006).

[2] Hare C, Poole KT (2014). The polarization of contemporary American politics. Polity.

[3] Axelrod, R., Daymude, J. J. & Forrest, S. Preventing extreme polarization of political attitudes. *Proc. Natl Acad. Sci. USA* **118**(50), e2102139118 (National Academy of Sciences, 2021). 10.1073/pnas.2102139118 PMC8685667 34876506

[4] Iyengar S, Lelkes Y, Levendusky M, Malhotra N, Westwood SJ (2019). The origins and consequences of affective polarization in the United States. Annu. Rev. Political Sci..

[5] Druckman JN, Klar S, Krupnikov Y, Levendusky M, Ryan JB (2021). Affective polarization, local contexts and public opinion in America. Nat. Hum. Behav..

[6] Vosoughi S, Roy D, Aral S (2018). The spread of true and false news online. Science.

[7] Juul, J. L. & Ugander, J. Comparing information diffusion mechanisms by matching on cascade size. *Proc. Natl Acad. Sci. USA* **118**(46), e2100786118 (National Academy of Sciences, 2021). 10.1073/pnas.2100786118 PMC8609637 34750252

[8] Guilbeault D, Centola D (2021). Topological measures for identifying and predicting the spread of complex contagions. Nat. Commun..

[9] Effing, R., Van Hillegersberg, J. & Huibers, T. Social media and political participation: are Facebook, Twitter and Youtube democratizing our political systems? In *International Conference on Electronic Participation* (eds Tambouris, E., Macintosh, A. & Bruijn, H.) 25–35 (Springer, 2011).

[10] Broersma M, Graham T (2012). Social media as beat: tweets as a news source during the 2010 British and Dutch elections. Journalism Pract..

[11] Metaxas PT, Mustafaraj E (2012). Social media and the elections. Science.

[12] Ceron, A., Curini, L. & Iacus, S. *Politics and Big Data: Nowcasting and Forecasting Elections with Social Media* (Taylor & Francis, 2016); 10.4324/9781315582733

[13] Bovet A, Morone F, Makse HA (2018). Validation of Twitter opinion trends with national polling aggregates: Hillary Clinton vs Donald Trump. Sci. Rep..

[14] Soares, F. B., Recuero, R. & Zago, G. Influencers in polarized political networks on Twitter. In *Proc. 9th International Conference on Social Media and Society* (eds Gruzd, A., Mai, P.), 168–177 (2018).

[15] Grover P, Kar AK, Dwivedi YK, Janssen M (2019). Polarization and acculturation in US Election 2016 outcomes – can Twitter analytics predict changes in voting preferences. Technol. Forecast. Soc. Change.

[16] Lee S, Xenos M (2019). Social distraction? Social media use and political knowledge in two US Presidential elections. Comput. Hum. Behav..

[17] Acharoui Z, Alaoui A, Ettaki B, Zerouaoui J, Dakkon M (2020). Identifying political influencers on YouTube during the 2016 Moroccan General Election. Procedia Comput. Sci..

[18] Suau-Gomila G, Pont-Sorribes C, Pedraza-Jiménez R (2020). Politicians or influencers? Twitter profiles of Pablo Iglesias and Albert Rivera in the Spanish general elections of 20-D and 26-J. Commun. Soc..

[19] Allcott H, Gentzkow M (2017). Social media and fake news in the 2016 election. J. Econ. Perspect..

[20] Shao C (2018). Anatomy of an online misinformation network. PLoS ONE.

[21] Bovet A, Makse HA (2019). Influence of fake news in Twitter during the 2016 US presidential election. Nat. Commun..

[22] Grinberg N, Joseph K, Friedland L, Swire-Thompson B, Lazer D (2019). Fake news on Twitter during the 2016 U.S. presidential election. Science.

[23] Ruths D (2019). The misinformation machine. Science.

[24] Machado, C., Kira, B., Narayanan, V., Kollanyi, B. & Howard, P. A study of misinformation in WhatsApp groups with a focus on the Brazilian presidential elections. In *Companion Proc. 2019 World Wide Web Conference* (eds Nivola, P. S. & Brady, D. W.), 1013–1019 (Rowman & Littlefield, 2019).

[25] Benkler, Y., Faris, R. & Roberts, H. Network propaganda: manipulation, disinformation, and radicalization in American politics (Oxford Univ. Press, 2018).

[26] Conover, M. et al. Political polarization on Twitter. In *Proc. International AAAI Conference on Web and Social Media* (eds Nicolov, N. & Shanahan, J. G.,) (PKP Publishing Services Network, 2011); https://ojs.aaai.org/index.php/ICWSM/article/view/14126

[27] Prior M (2013). Media and political polarization. Annu. Rev. Polit. Sci..

[28] Mocanu D, Rossi L, Zhang Q, Karsai M, Quattrociocchi W (2015). Collective attention in the age of (mis)information. Comput. Hum. Behav..

[29] Barberá P, Jost JT, Nagler J, Tucker JA, Bonneau R (2015). Tweeting from left to right: is online political communication more than an echo chamber?. Psychol. Sci..

[30] Bessi A (2016). Homophily and polarization in the age of misinformation. Eur. Phys. J. Spec. Top..

[31] Vaccari, C. et al. Of echo chambers and contrarian clubs: exposure to political disagreement among German and Italian users of Twitter. *Soc. Media Soc.* **2**, 10.1177/2056305116664221 (2016).

[32] Bessi, A. et al. Users polarization on Facebook and Youtube. *PLoS ONE* **11**(8):e0159641; 10.1371/journal.pone.0159641 (2016). 10.1371/journal.pone.0159641 PMC4994967 27551783

[33] Lelkes Y, Sood G, Iyengar S (2017). The hostile audience: the effect of access to broadband internet on partisan affect. Am. J. Polit. Sci..

[34] Bail CA (2018). Exposure to opposing views on social media can increase political polarization. Proc. Natl Acad. Sci. USA.

[35] Cinelli M, De Francisci Morales G, Galeazzi A, Quattrociocchi W, Starnini M (2021). The echo chamber effect on social media. Proc. Natl Acad. Sci. USA.

[36] McCarty N (2019). Polarization: What Everyone Needs to Know.

[37] Galston, W. A. & Nivola, P. S. Delineating the problem *in Red and Blue Nation: Characteristics and Causes of America’s Polarized Politics* (eds Nivola, P. S. & Brady, D. W.) **1**(1):1–46 (Brookings Institute Press, Washington D.C., 2006).

[38] Abramowitz, A. I. & Fiorina, M. P. (2017, July 14). Polarized or sorted? Just what’s wrong with our politics, anyway. *Amer. Interest.* Retrieved February 19, 2023, from https://www.the-american-interest.com/2013/03/11/polarized-or-sorted-just-whats-wrong-with-our-politics-anyway/

[39] Fiorina MP, Abrams SJ (2008). Political polarization in the American public. Annu. Rev. Polit. Sci..

[40] Layman GC, Carsey TM, Horowitz JM (2006). Party polarization in American politics: characteristics, causes, and consequences. Annu. Rev. Polit. Sci..

[41] Mason L (2015). ‘I disrespectfully agree’: the differential effects of partisan sorting on social and issue polarization. Am. J. Polit. Sci..

[42] Brown JR, Enos RD (2021). The measurement of partisan sorting for 180 million voters. Nat. Hum. Behav..

[43] Druckman JN, Levendusky MS (2019). What do we measure when we measure affective polarization?. Public Opin. Q..

[44] Dalton RJ (2021). Modeling ideological polarization in democratic party systems. Elect. Stud..

[45] Bakshy E, Messing S, Adamic LA (2015). Exposure to ideologically diverse news and opinion on Facebook. Science.

[46] Efthimion PG, Payne S, Proferes N (2018). Supervised machine learning bot detection techniques to identify social Twitter bots. SMU Data Sci. Rev..

[47] Metaxas, P. et al. What do retweets indicate? Results from user survey and meta-review of research. In *Proc. Int. AAAI Conf. Web Soc. Media*, **9** (ed. Quercia, D.)(PKP Publishing Services Network, 2015).

[48] Morone F, Makse HA (2015). Influence maximization in complex networks through optimal percolation. Nature.

[49] Barberá P (2015). Birds of the same feather tweet together: Bayesian ideal point estimation using Twitter data. Polit. Anal..

[50] Benzécri, J.-P. et al. *L’analyse des données*, Vol. 2 (Dunod, 1973).

[51] Nenadic O, Greenacre M (2007). Correspondence analysis in R, with two-and three-dimensional graphics: the ca package. J. Statist. Softw..

[52] Hartigan JA, Hartigan PM (1985). The dip test of unimodality. Ann. Stat..

[53] Bozarth, L., Saraf, A. & Budak, C. Higher ground? How groundtruth labeling impacts our understanding of fake news about the 2016 US presidential nominees. In *Proc. Int. AAAI Conf. Web Soc. Media*, **14** (ed. De Choudhury, M.) 48–59 (PKP Publishing Services Network, 2020).

[54] Main, T. J. *The Rise of the Alt-Right* (Brookings Institution Press, 2018).

[55] Stefanov, P., Darwish, K., Atanasov, A. & Nakov, P. Predicting the topical stance and political leaning of media using tweets. In *Proc. 58th Annual Meeting of the Association for Computational Linguistics* (eds Jurafsky, D, Chai, J., Schluter, N. & Tetreault, J.), 527–537 (Association for Computational Linguistics, 2020).

[56] Cinelli M (2020). The COVID-19 social media infodemic. Sci. Rep..

[57] Desai S. & Oehrli, *J. A., ‘Fake News,’ Lies and Propaganda: How to Sort Fact from Fiction*, accessed 21 July 2022; https://guides.lib.umich.edu/c.php?g=637508&p=4462444 (2022).

[58] Kempe, D., Kleinberg, J. & Tardos, É. Maximizing the spread of influence through a social network. In *Proc. of the 9th ACM SIGKDD International Conference on Knowledge Discovery and Data Mining* (ed. Senator, T.) 137–146 (ACM Press, New York, NY, 2003).

[59] Bakshy, E., Hofman, J. M., Mason, W. A. & Watts, D. J. Identifying influencers on Twitter. In *4th ACM International Conference on Web Search and Data Mining (WSDM)* (ed. King, I.), **2** (ACM, New York, NY, 2011).

[60] Blondel VD, Guillaume J-L, Lambiotte R, Lefebvre E (2008). Fast unfolding of communities in large networks. J. Stat. Mech. Theory Exp..

[61] Brandes U (2007). On modularity clustering. IEEE Trans. Knowl. Data Eng..

