# Supplementary material for: Political polarization of news media and influencers on Twitter in the 2016 and 2020 US presidential elections
Source: Nat Hum Behav. 2023 Mar 13;7(6):904–16. doi: 10.1038/s41562-023-01550-8 (PMC10289895; doi:10.1038/s41562-023-01550-8)
Supplement: Supplementary file 1 — Supplementary text, figs. 1–10 and tables 1–10. [file 41562_2023_1550_MOESM1_ESM.pdf]

nature human behaviour

Article

https://doi.org/10.1038/s41562-023-01550-8

Political polarization of news media and influencers on Twitter in the 2016 and 2020 US presidential elections

In the format provided by the authors and unedited

This PDF file includes:

Supplementary Text

Supplementary Figures 1 to 10

Supplementary Tables 1 to 10

Supplementary Text

Definitions of Polarization

We acknowledge that political scientists distinguish multiple types of polarization [36, 37, 38, 39, 40, 41, 42, 43, 44]: affective polarization (the penchant for one partisan political group to experience animus toward an opposing partisan group), policy polarization (extreme differences of opinion on highly salient issues), partisan polarization (a substantive and affective division based on identification with opposing political parties), ideological polarization (a substantive and affective division based on identification with opposing ideological camps, e.g., liberals versus conservatives), and geographic polarization (the regional alignment of opinions, e.g., "red state/blue state"). Furthermore, each of these five types of polarization can, in turn, be classified by level: elite polarization among political officials and pundits, media polarization among news organizations, and voter polarization among the underlying population as usually measured by exit polls and opinion surveys. In the main manuscript, we seek to explore polarization to quantify the various ways the Twitter communities disseminate news. Accordingly, we opt to define polarization in the main manuscript as the growth in ideological separation between Twitter users as characterized by the political alignment of the content they propagate.

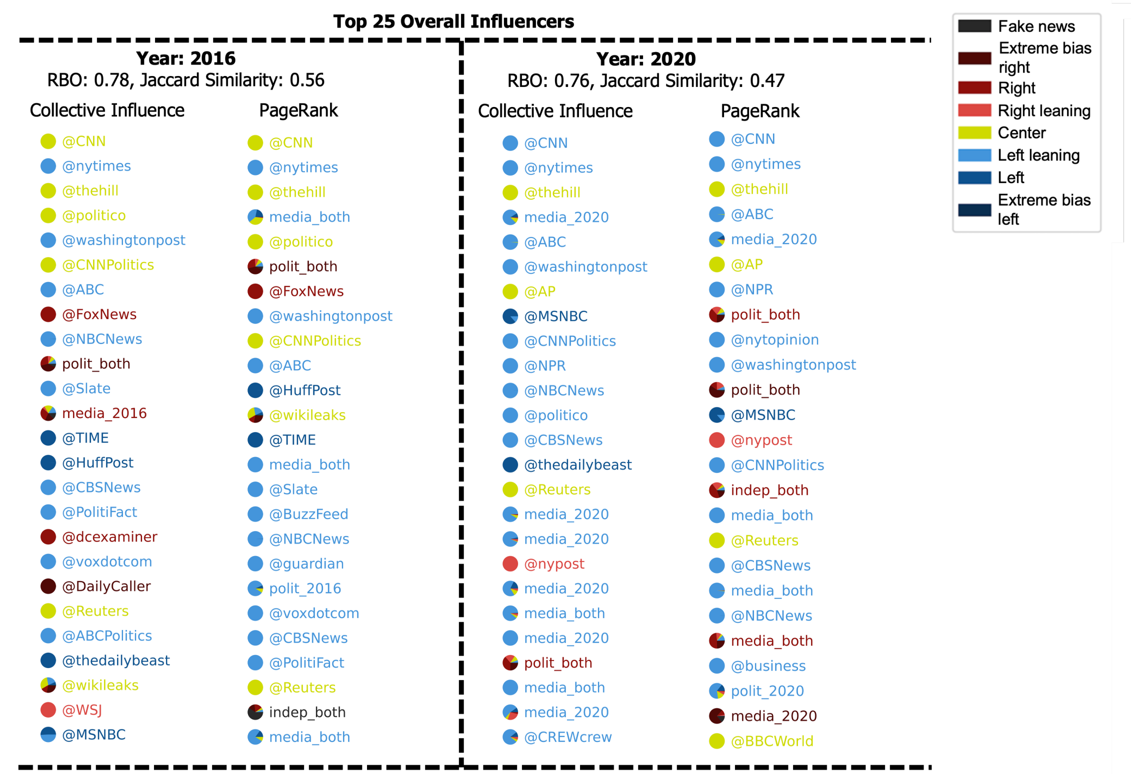

Supplementary Figure 1. Top 25 influencers overall, extracted using Collective Influence and PageRank. Influencers are ordered by rank, starting from rank 1 at the top, as determined by their CI_{out} score or their PageRank score. This was done for each year by generating an overall retweet network that combined all news media category networks into one, with influencers being extracted from the result and ranked in decreasing order of centrality score magnitude. For CI, this network is unweighted, while for PR is weighted. Ranked Biased Overlap (RBO, where $p = 0.98$) and Jaccard Similarity were used to compare the two resultant ranked lists of each year. The neighboring pie chart slices represent the fraction of each news media category content that the influencer propagated, with the influencer username or alias being colored with the color of the largest slice (indicating the news media category in which they disseminated the most information).

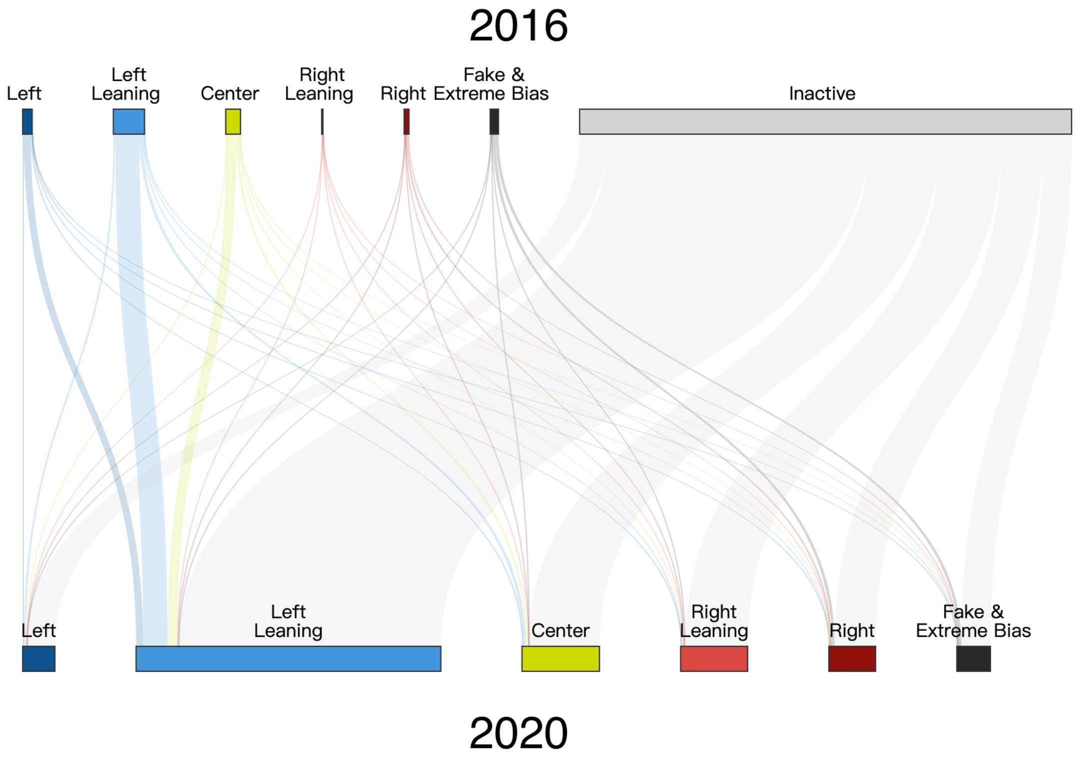

Supplementary Figure 2. Shifts of users across news media categories from 2016 to 2020 including the flow of inactive (or non-existent) users in 2016 to active news media categories in 2020. See Tab. 6 for the raw numbers used to generate this figure.

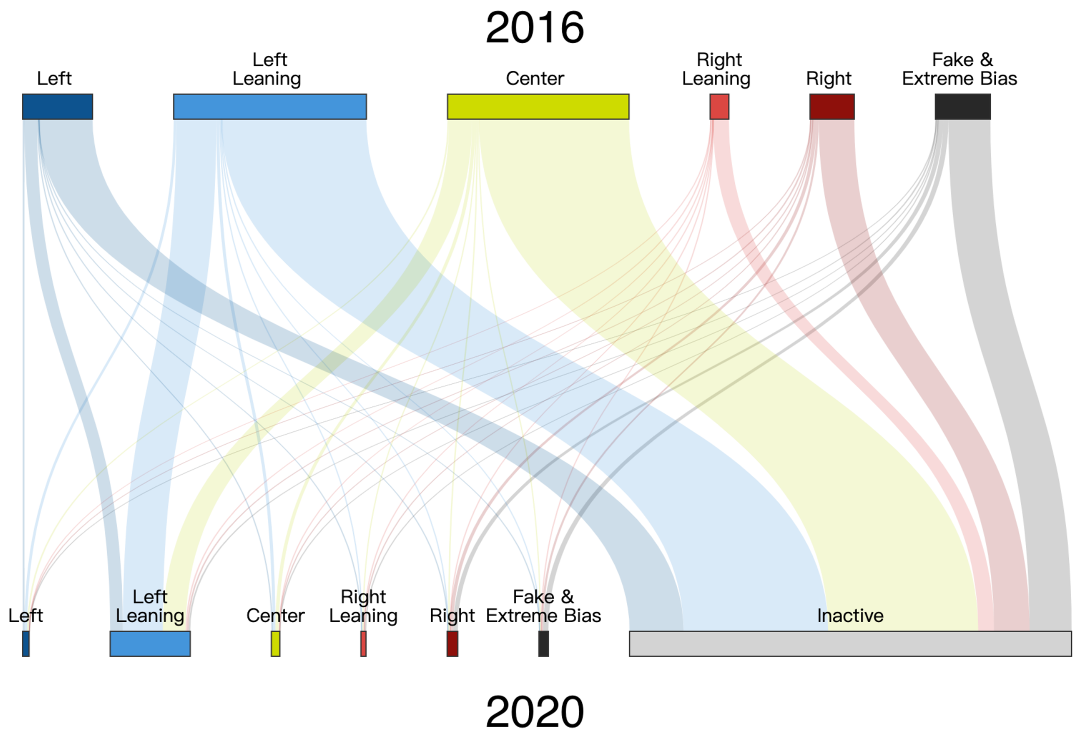

Supplementary Figure 3. Shifts of users across news media categories from 2016 to 2020 including the flow of active users from different news media categories in 2020 to inactivity in 2020 due to banning, account deletion, or overall non-participation. See Tab. 6 for the raw numbers used to generate this figure.

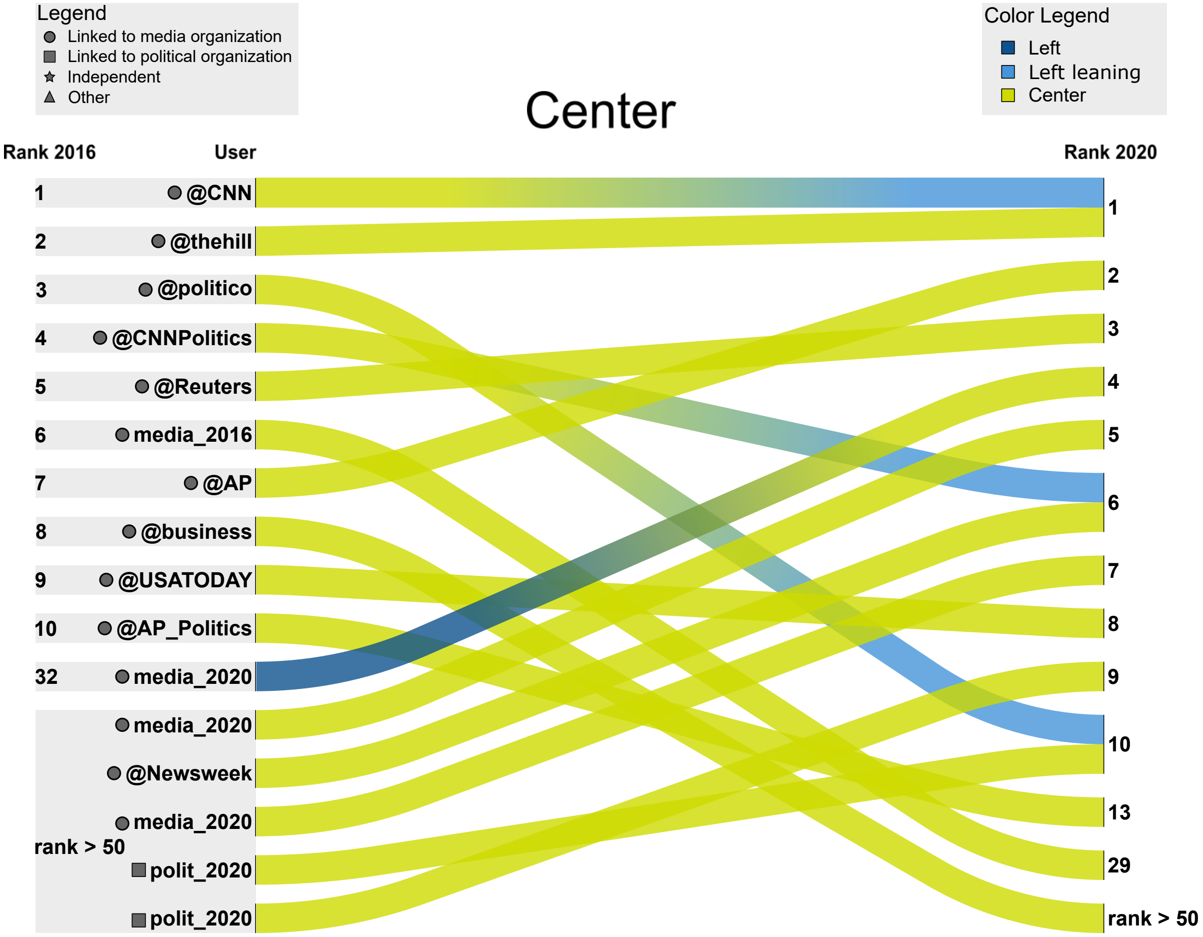

Supplementary Figure 4. Change in rankings 2016-2020, Center Bias. Outlines the change in the ranks of the top 10 center bias users from 2016 and 2020, ranked by CI influence. Each flow connects the best ranking for a user in 2016, whose rank is displayed to the left of the username or alias, to their rank in 2020. The colors of the lines match the bias of the users best ranking, and gradients represent a change in the bias classification of their best ranking.

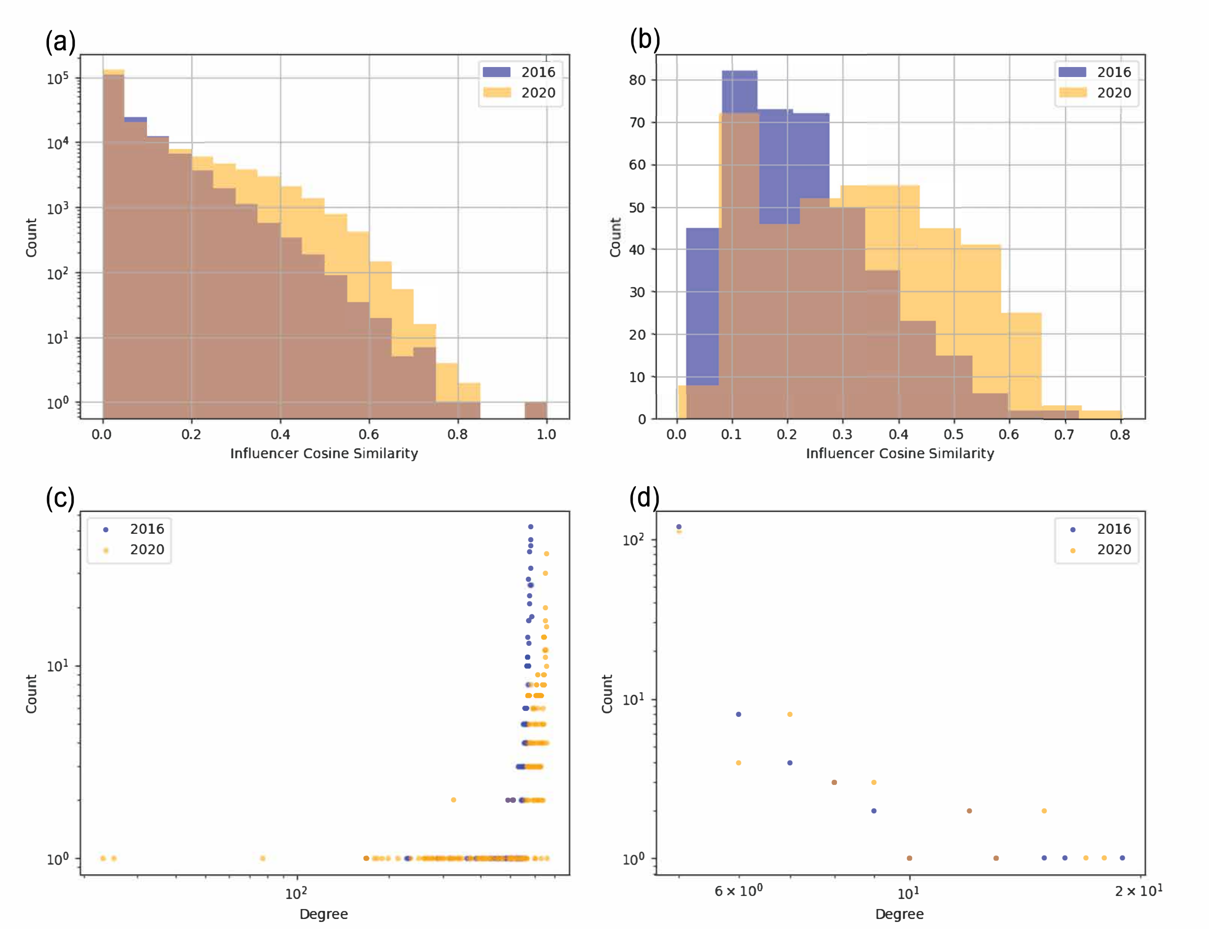

Supplementary Figure 5. Weight and degree distributions for the influencer similarity networks and Figure 5. Figure 5 a shows the distribution of the influencer cosine similarity weights for the 2016 and 2020 full similarity networks generated at the beginning of the "Polarization among Twitter users" subsection in the main manuscript. Figure 5 b shows the same distributions but only for the visible edges of the subsampled networks in Figure 5. Figure 5 c and d show the degree distribution for the full similarity networks and for the visible edges of Figure 5, respectively.

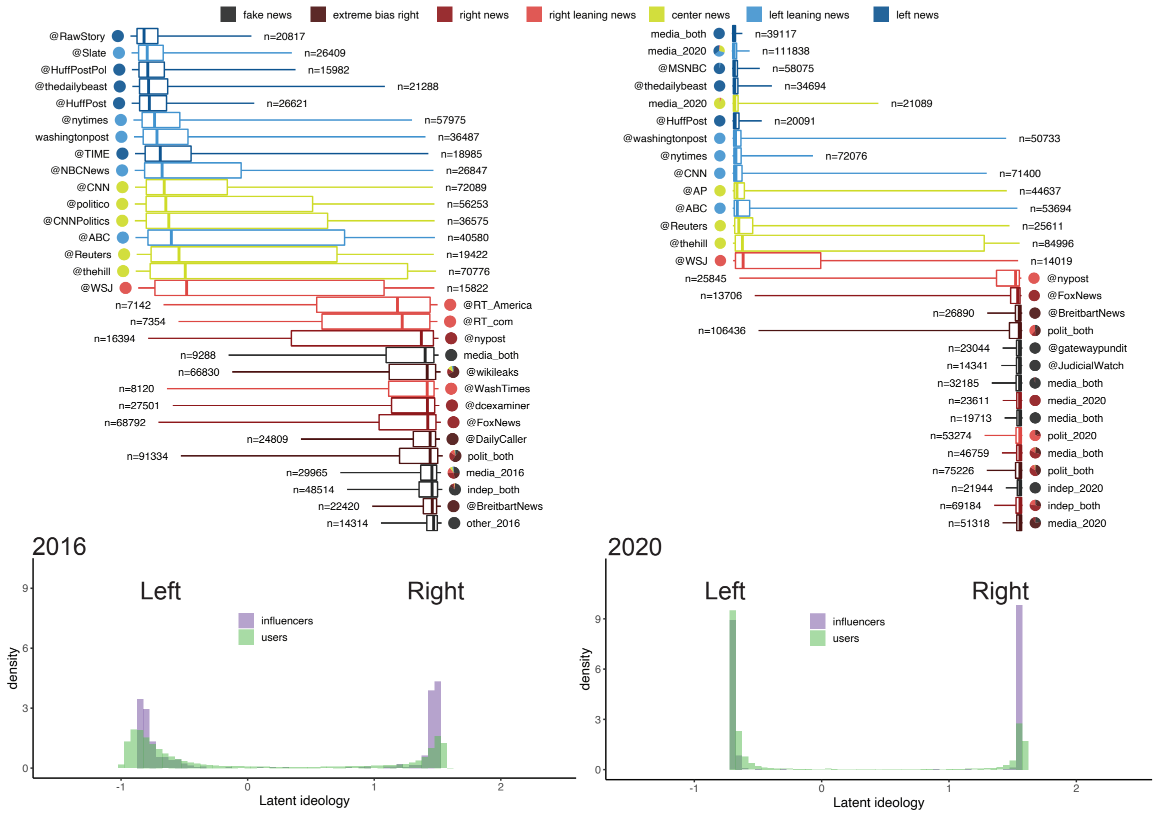

Supplementary Figure 6. Latent ideology scale of influencers and their retweeters in 2016 (left) and 2020 (right) using only users active in both years. The latent ideology of the top 5 influencers of each category is shown as a box plot representing the distribution of the ideology of the users having retweeted them. The distribution of the ideology estimates of the users is shown in green and the distribution of the ideology estimates of the top 100 influencers of each news category (computed as the median of the ideology of their retweeters) is displayed in purple. Box plots indicate the median and the 25% and 75% percentiles of the distributions with whiskers indicating the 5% and 95% percentiles. The sample size used for the computation of each box plot is reported to their side. Pie charts next to the influencers' names represent the news categories they belong to (weighted by their respective CI ranks in each category). Hartigans' dip test for unimodality (one-sided) applied to the user distribution is $D = 0.094$ (95% confidence interval $\mathrm{CI}_{95\%} = [0.0934, 0.0947]$, $p < 2.2 \times 10^{-16}$) in 2016 and $D = 0.117$ ($\mathrm{CI}_{95\%} = [0.1166, 0.1178]$, $p < 2.2 \times 10^{-16}$) in 2020. The test statistics for the influencer distribution is $D = 0.178$ ($\mathrm{CI}_{95\%} = [0.1616, 0.1979]$, $p < 2.2 \times 10^{-16}$) in 2016 and $D = 0.214$ ($\mathrm{CI}_{95\%} = [0.1952, 0.2336]$, $p < 2.2 \times 10^{-16}$) in 2020.

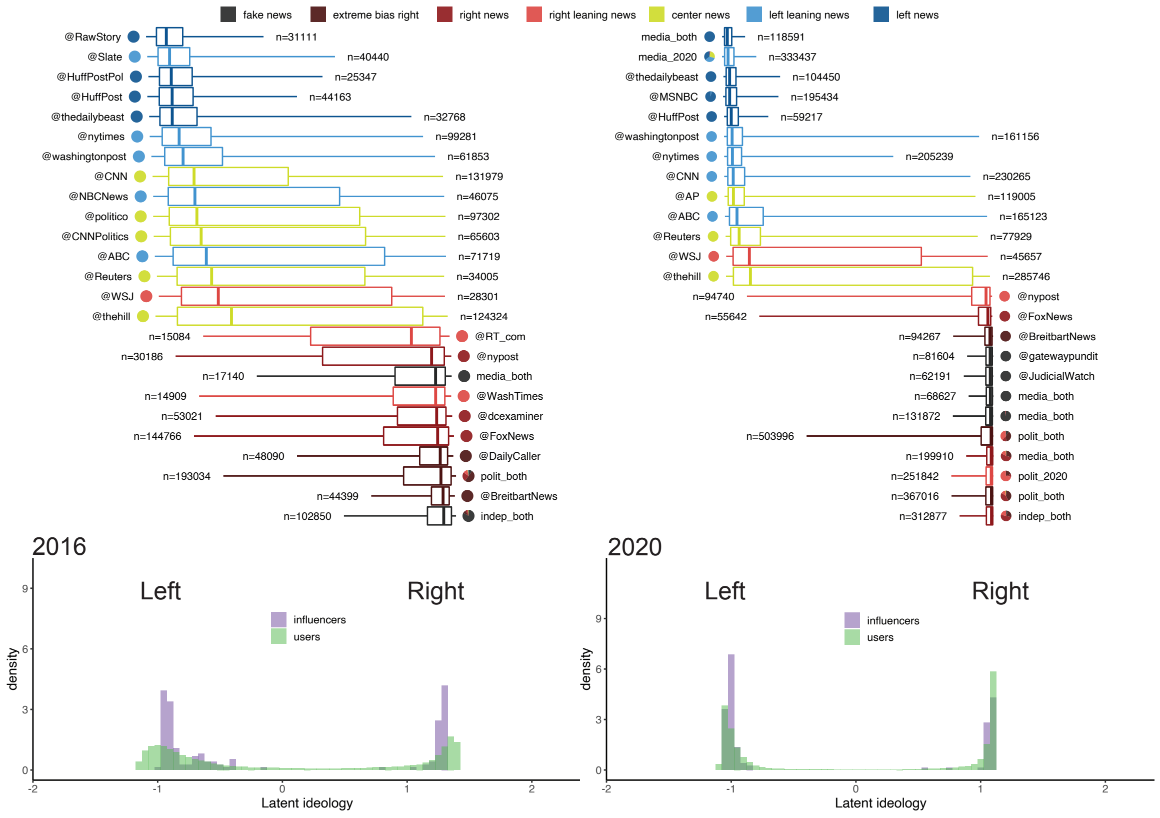

Supplementary Figure 7. Latent ideology scale of influencers and their retweeters in 2016 (left) and 2020 (right) using only influencers active in both years. The latent ideology of the top 5 influencers of each category is shown as a box plot representing the distribution of the ideology of the users having retweeted them. The distribution of the ideology estimates of the users is shown in green and the distribution of the ideology estimates of the top 100 influencers of each news category (computed as the median of the ideology of their retweeters) is displayed in purple. Box plots indicate the median and the 25% and 75% percentiles of the distributions with whiskers indicating the 5% and 95% percentiles. The sample size used for the computation of each box plot is reported to their side. Pie charts next to the influencers' names represent the news categories they belong to (weighted by their respective CI ranks in each category). Hartigans' dip test for unimodality (one-sided) applied to the user distribution is $D = 0.107$ (95% confidence interval $\mathrm{CI}_{95\%} = [0.1065, 0.1076]$, $p < 2.2 \times 10^{-16}$) in 2016 and $D = 0.183$ ($\mathrm{CI}_{95\%} = [0.1825, 0.1834]$, $p < 2.2 \times 10^{-16}$) in 2020. The test statistics for the influencer distribution is $D = 0.163$ ($\mathrm{CI}_{95\%} = [0.1290, 0.1951]$, $p < 2.2 \times 10^{-16}$) in 2016 and $D = 0.173$ ($\mathrm{CI}_{95\%} = [0.1376, 0.2122]$, $p < 2.2 \times 10^{-16}$) in 2020.

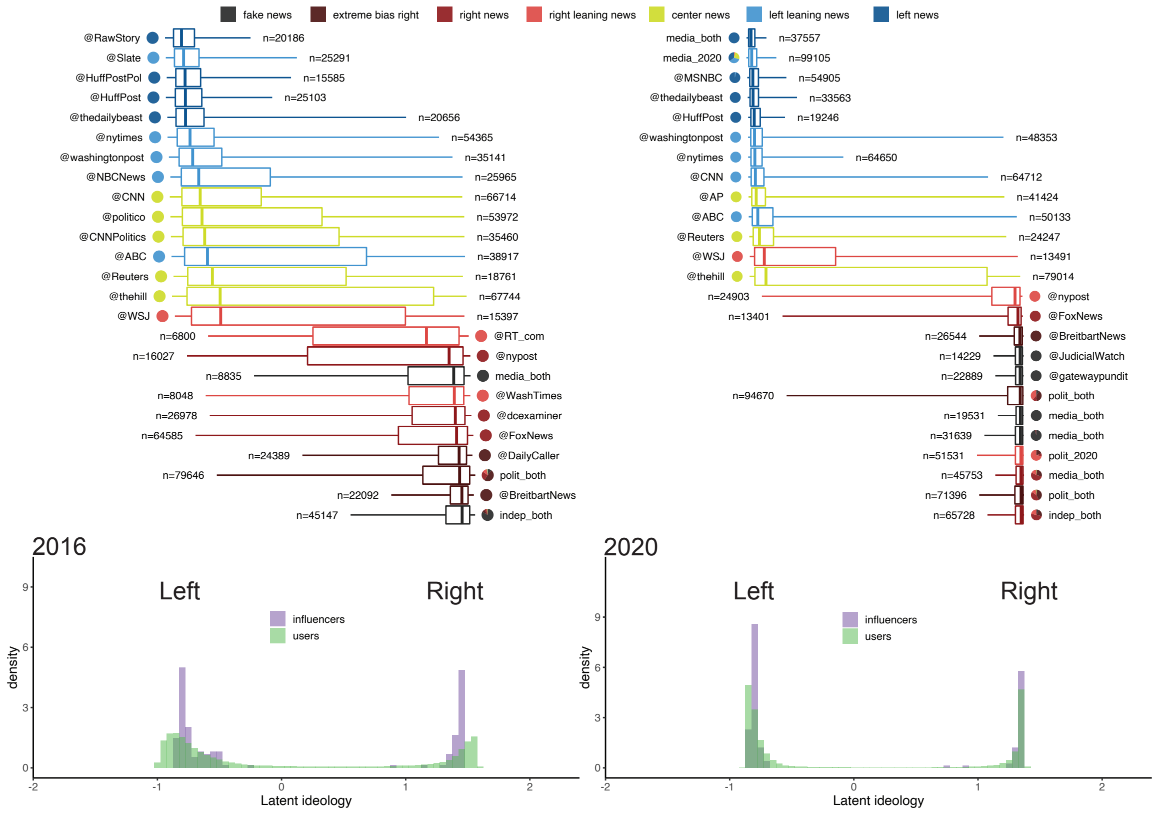

Supplementary Figure 8. Latent ideology scale of influencers and their retweeters in 2016 (left) and 2020 (right) using only users and influencers active in both years. The latent ideology of the top 5 influencers of each category is shown as a box plot representing the distribution of the ideology of the users having retweeted them. The distribution of the ideology estimates of the users is shown in green and the distribution of the ideology estimates of the top 100 influencers of each news category (computed as the median of the ideology of their retweeters) is displayed in purple. Box plots indicate the median and the 25% and 75% percentiles of the distributions with whiskers indicating the 5% and 95% percentiles. The sample size used for the computation of each box plot is reported to their side. Pie charts next to the influencers' names represent the news categories they belong to (weighted by their respective CI ranks in each category). Hartigans' dip test for unimodality (one-sided) applied to the user distribution is $D = 0.095$ (95% confidence interval $\mathrm{CI}_{95\%} = [0.0940, 0.0955]$, $p < 2.2 \times 10^{-16}$) in 2016 and $D = 0.140$ ($\mathrm{CI}_{95\%} = [0.1390, 0.1406]$, $p < 2.2 \times 10^{-16}$) in 2020. The test statistics for the influencer distribution is $D = 0.164$ ($\mathrm{CI}_{95\%} = [0.1314, 0.2034]$, $p < 2.2 \times 10^{-16}$) in 2016 and $D = 0.171$ ($\mathrm{CI}_{95\%} = [0.1379, 0.2086]$, $p < 2.2 \times 10^{-16}$) in 2020.

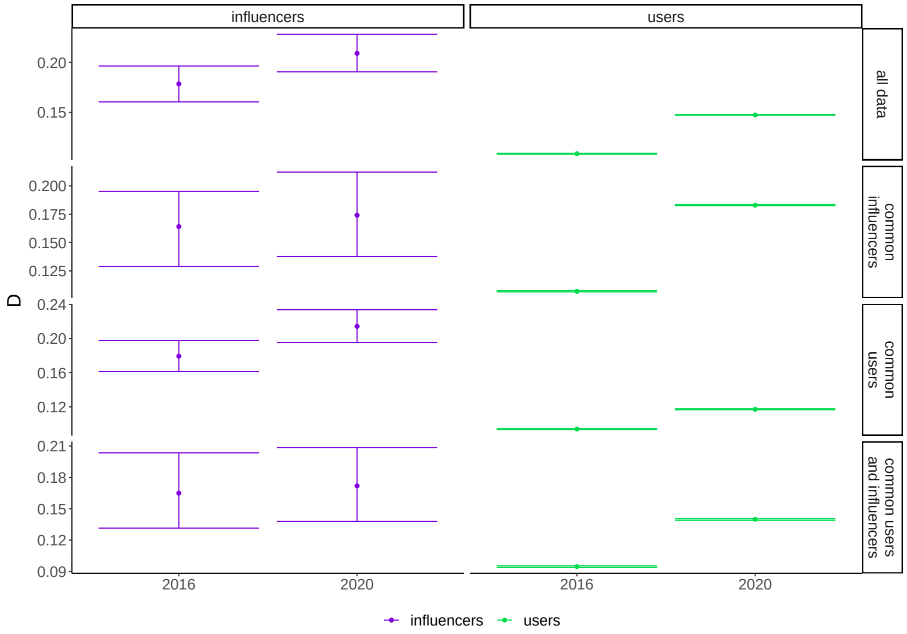

Supplementary Figure 9. Hartigans' dip test values for ideology distribution of users and influencers when considering all users and influencers or only influencers or users present in 2016 and 2020. Mean and 95% CI error bars are obtained by bootstrap with n =1000 runs for each dataset and Bias-corrected and accelerated confidence intervals method. The numerical values are reported in Table 10.

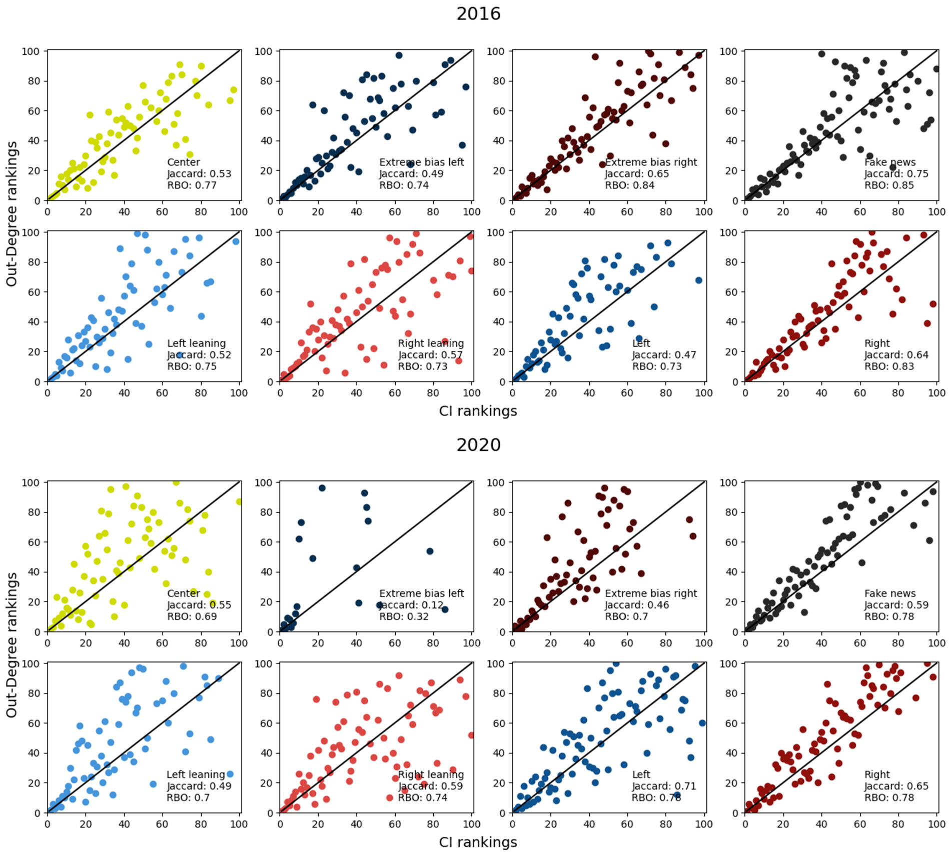

Supplementary Figure 10. Comparison of top 100 rankings generated by the PageRank algorithm and by the Collective Influence (CI) algorithm using the 2016 and 2020 retweet networks. CI operates on the unweighted, directed retweet networks while PR operates on a weighted, directed version of the retweet networks, where a retweet edge is weighted by the number of times the node was retweeted. Ranked Bias Overlap (RBO) [62] and Jaccard Similarity are computed over the two top 100 lists, shown below their respective news category labels. For this analysis, RBO's weight parameter p is set to 0.98. The most of RBO values are above 0.7 indicating a high agreement of the two rankings, especially for the top ranked users. The only network that shows a poor agreement between the rankings is the extreme-bias left network of 2020. This may be explained by the small size and low average degree of the network compared to networks of other categories (see Tab. 4).

	Fake news		Extreme bias right		Right	
	hostnames	N	hostnames	N	hostnames	N
1	thegatewaypundit.com	761 756	breitbart.com	1 854 920	foxnews.com	1 122 732
2	truthfeed.com	554 955	dailycaller.com	759 504	dailymail.co.uk	474 846
3	infowars.com	478 872	americanthinker.com	179 696	washingtonexaminer.com	462 769
4	therealstrategy.com	241 354	wnd.com	141 336	nypost.com	441 648
5	conservativetribune.com	212 273	freebeacon.com	129 077	bizpacreview.com	170 770
6	zerohedge.com	186 706	newsninja2012.com	127 251	nationalreview.com	164 036
7	rickwells.us	78 736	hannity.com	114 221	lifezette.com	139 257
8	departed.co	72 773	newsmax.com	94 882	redstate.com	105 912
9	thepoliticalinsider.com	66 426	endingthefed.com	88 376	allenbwest.com	104 857
10	therightscoop.com	63 852	truepundit.com	84 967	theconservativetreehouse.com	102 515
11	teaparty.org	48 757	westernjournalism.com	77 717	townhall.com	102 408
12	usapoliticsnow.com	46 252	dailywire.com	67 893	investors.com	102 295
13	clashdaily.com	45 970	newsbusters.org	60 147	theblaze.com	99 029
14	thefederalistpapers.org	45 831	ilovemyfreedom.org	54 772	theamericanmirror.com	91 538
15	redflagnews.com	45 423	100percentfedup.com	54 596	ijr.com	71 558
16	thetruthdivision.com	44 486	pjmedia.com	46 542	judicialwatch.org	70 543
17			weaselzippers.us	45 199	thefederalist.com	55 835
18					hotair.com	55 431
19					conservativereview.com	54 307
20					weeklystandard.com	50 707

	Right leaning		Center		Left leaning	
	hostnames	N	hostnames	N	hostnames	N
1	wsj.com	310 416	cnn.com	2 291 736	nytimes.com	1 811 627
2	washingtontimes.com	208 061	thehill.com	1 200 123	washingtonpost.com	1 640 088
3	rt.com	157 474	politico.com	1 173 717	nbcnews.com	512 056
4	realclearpolitics.com	128 417	usatoday.com	326 198	abcnews.go.com	467 533
5	telegraph.co.uk	82 118	reuters.com	283 962	theguardian.com	439 580
6	forbes.com	64 186	bloomberg.com	266 662	vox.com	369 789
7	fortune.com	57 644	businessinsider.com	239 423	slate.com	279 438
8			apnews.com	198 140	buzzfeed.com	278 642
9			observer.com	128 043	cbsnews.com	232 889
10			fivethirtyeight.com	124 268	politifact.com	198 095
11			bbc.com	118 176	latimes.com	190 994
12			ibtimes.com	72 424	nydailynews.com	188 769
13			bbc.co.uk	71 941	theatlantic.com	177 637
14					mediaite.com	152 877
15					newsweek.com	149 490
16					npr.org	142 143
17					independent.co.uk	127 689
18					cnb.cx	87 094
19					hollywoodreporter.com	84 997

	Left		Extreme bias left	
	hostnames	N	hostnames	N
1	huffingtonpost.com	1 057 518	dailynewsbin.com	189 257
2	thedailybeast.com	378 931	bipartisanreport.com	119 857
3	dailykos.com	324 351	bluenationreview.com	75 455
4	rawstory.com	297 256	crooksandliars.com	73 615
5	politicususa.com	293 419	occupydemocrats.com	73 143
6	time.com	252 468	shareblue.com	50 880
7	motherjones.com	210 280	usuncut.com	27 653
8	talkingpointsmemo.com	199 346		
9	msnbc.com	177 090		
10	mashable.com	173 129		
11	salon.com	172 807		
12	thinkprogress.org	172 144		
13	newyorker.com	171 102		
14	mediamatters.org	152 160		
15	nymag.com	121 636		
16	theintercept.com	109 591		
17	thenation.com	54 661		
18	people.com	47 942		

Supplementary Table 1. Hostnames in each news media category in 2016. We also show the number (N) of tweets with a URL pointing toward each hostname. Tweets with several URLs are counted multiple times. Reproduced from [21].

	Fake news		Extreme bias right		Right	
	hostnames	N	hostnames	N	hostnames	N
1	thegatewaypundit.com	1 883 852	breitbart.com	2 192 997	foxnews.com	3 136 578
2	hannity.com	428 483	dailymail.co.uk	600 523	dailycaller.com	771 765
3	waynedupree.com	258 838	bongino.com	346 103	washingtonexaminer.com	717 017
4	judicialwatch.org	233 085	thenationalpulse.com	215 017	justthenews.com	689 725
5	truepundit.com	176 647	freebeacon.com	197 092	thefederalist.com	687 091
6	zerohedge.com	165 960	newsmax.com	192 924	dailywire.com	396 233
7	davidharrisjr.com	150 887	pjmedia.com	123 338	theepochtimes.com	288 656
8	politicalflare.com	145 838	newsbusters.org	71 008	nationalreview.com	283 172
9	djhjmedia.com	112 049	therightscoop.com	66 676	saraacarter.com	267 237
10	rumble.com	101 979	americanthinker.com	59 142	townhall.com	256 631
11	theconservativetreehouse.com	99 716			theblaze.com	191 515
12	oann.com	97 325			thepostmillennial.com	181 674
13	thedcpatriot.com	90 209			westernjournal.com	165 914
14	washingtonnews.today	79 314			redstate.com	144 010
15	rightwingtribune.com	58 442			thegreggjarrett.com	139 749
16	rt.com	54 985			bizpacreview.com	97 375
17	wnd.com	54 929			twitchy.com	95 401
18	gellerreport.com	54 277			trendingpolitics.com	92 094
19	nationalfile.com	52 393			lifenews.com	90 064
20	summit.news	49 539				

	Right leaning		Center		Left leaning	
	hostnames	N	hostnames	N	hostnames	N
1	nypost.com	1 701 531	thehill.com	2 256 888	nytimes.com	6 775 402
2	wsj.com	887 537	apnews.com	1 182 504	washingtonpost.com	6 438 506
3	forbes.com	748 636	usatoday.com	993 957	cnn.com	5 577 352
4	washingtontimes.com	408 349	businessinsider.com	773 328	politico.com	2 290 755
5	foxbusiness.com	212 742	newsweek.com	756 820	nbcnews.com	2 231 564
6	thebulwark.com	175 417	reuters.com	746 033	theguardian.com	1 116 515
7	marketwatch.com	96 626	bbc.com	296 098	theatlantic.com	1 046 475
8	realclearpolitics.com	93 120	economist.com	123 939	abcnews.go.com	1 042 419
9	detroitnews.com	77 223	fivethirtyeight.com	101 824	npr.org	871 571
10	dallasnews.com	75 910	ft.com	91 524	bloomberg.com	767 059
11	rasmussenreports.com	58 712	foreignpolicy.com	87 729	cbsnews.com	747 442
12	chicagotribune.com	56 974	factcheck.org	79 456	cnbc.com	649 041
13	jpost.com	55 223	news.sky.com	78 372	axios.com	621 609
14					msn.com	613 127
15					news.yahoo.com	586 724
16					independent.co.uk	513 765
17					latimes.com	451 878
18					citizensforethics.org	382 101
19					buzzfeednews.com	369 962

	Left		Extreme bias left	
	hostnames	N	hostnames	N
1	rawstory.com	2 148 200	occupydemocrats.com	18 151
2	msnbc.com	1 606 071	lancastercourier.com	5815
3	thedailybeast.com	1 404 756	deepleftfield.info	5753
4	huffpost.com	1 121 642	tplnews.com	4022
5	politicususa.com	671 043	bipartisanreport.com	3243
6	palmerreport.com	434 503	bossip.com	2287
7	motherjones.com	424 106	polipace.com	586
8	vox.com	420 613		
9	vanityfair.com	352 964		
10	nymag.com	320 049		
11	newyorker.com	288 409		
12	dailykos.com	288 384		
13	slate.com	250 942		
14	salon.com	229 583		
15	rollingstone.com	190 828		
16	thenation.com	130 272		
17	alternet.org	126 788		
18	theintercept.com	104 153		

Supplementary Table 2. Hostnames in each news media category in 2020. We also show the number (N) of tweets with a URL pointing toward each hostname. Tweets with several URLs are counted multiple times.

2016								
	N_t	p_t	N_u	p_u	N_t/N_u	$p_{t,n/o}$	$p_{u,n/o}$	$N_{t,n/o}/N_{u,n/o}$
Fake news	2 991 073	0.10	68 391	0.03	43.73	0.19	0.07	124.22
Extreme bias right	3 969 639	0.13	131 346	0.06	30.22	0.09	0.05	56.73
Right	4 032 284	0.13	194 229	0.08	20.76	0.11	0.07	33.77
Right leaning	1 006 746	0.03	64 771	0.03	15.54	0.18	0.09	31.56
Center	6 322 257	0.21	600 546	0.26	10.53	0.20	0.05	38.10
Left leaning	7 491 344	0.24	903 689	0.39	8.29	0.14	0.06	19.16
Left	4 353 999	0.14	327 411	0.14	13.30	0.14	0.07	26.16
Extreme bias left	609 503	0.02	19 423	0.01	31.38	0.06	0.03	74.21

2020								
	N_t	p_t	N_u	p_u	N_t/N_u	$p_{t,n/o}$	$p_{u,n/o}$	$N_{t,n/o}/N_{u,n/o}$
Fake news	4 348 747	0.06	99 020	0.03	43.92	0.01	0.01	81.77
Extreme bias right	4 064 820	0.06	107 250	0.03	37.90	0.02	0.01	73.62
Right	8 691 901	0.12	382 358	0.10	22.73	0.02	0.01	44.52
Right leaning	4 648 000	0.06	288 207	0.08	16.13	0.02	0.01	23.35
Center	7 568 472	0.10	398 241	0.11	19.00	0.03	0.02	33.96
Left leaning	33 093 267	0.45	2 136 830	0.59	15.49	0.03	0.02	22.85
Left	10 513 306	0.14	237 685	0.07	44.23	0.03	0.02	73.42
Extreme bias left	39 857	0.00	887	0.00	44.93	0.05	0.02	82.59

Supplementary Table 3. Tweet and user volume corresponding to each news media category on Twitter between June 1st until election day in 2016 (top) and 2020 (bottom). Number, N_{t}, and proportion, p_{t}, of tweets with a URL pointing to a website belonging to one of the news media categories. Number, N_{u}, and proportion, p_{u}, of unique users in each category. Users are classified in the category where they posted the largest number of tweets. Ties are randomly assigned. Proportion of tweets sent by non-official clients, $p_{\mathrm{t,n/o}}$, proportion of users having sent at least one tweet from a non-official client, $p_{\mathrm{u,n/o}}$, and average number of tweets per user sent from non-official clients, $N_{\mathrm{t,n/o}}/N_{u,n/o}$. 2016 data adapted from [21].

	News media category	Nodes	Edges	$\langle k \rangle$	max(k_{out})	max(k_{in})	$\sigma(k_{out})/\langle k \rangle$	$\sigma(k_{in})/\langle k \rangle$
2016	Fake news	175,605	1,143,083	6.51	42,468	1232	32 ± 4	2.49 ± 0.06
	Extreme bias right	249,659	1,637,927	6.56	51,845	588	36 ± 6	2.73 ± 0.03
	Right	345,644	1,797,023	5.20	86,454	490	44 ± 11	2.70 ± 0.04
	Right leaning	216,026	495,307	2.29	32,653	129	45 ± 11	1.72 ± 0.02
	Center	864,733	2,501,037	2.89	229,751	512	75 ± 39	2.69 ± 0.06
	Left leaning	1,043,436	3,570,653	3.42	145,047	843	59 ± 19	3.38 ± 0.10
	Left	536,903	1,801,658	3.36	58,901	733	47 ± 12	3.50 ± 0.08
	Extreme bias left	78,911	277,483	3.52	23,168	648	33 ± 6	2.49 ± 0.08
2020	Fake news	367,487	1,861,620	5.06	90,125	292	59 ± 11	2.05 ± 0.02
	Extreme bias right	445,776	2,008,760	4.50	89,902	313	60 ± 16	2.09 ± 0.02
	Right	674,935	4,452,861	6.59	109,053	607	54 ± 9	2.43 ± 0.03
	Right leaning	882,552	3,203,999	3.63	115,302	298	59 ± 16	1.86 ± 0.02
	Center	1,163,610	4,461,011	3.83	276,289	709	65 ± 29	2.37 ± 0.04
	Left leaning	2,355,587	17,461,102	7.41	325,726	1,564	63 ± 20	3.62 ± 0.05
	Left	819,684	4,688,119	5.71	175,841	1,042	57 ± 14	2.68 ± 0.04
	Extreme bias left	21,411	26,888	1.25	5,755	27	41 ± 3	0.60 ± 0.01

Supplementary Table 4. Retweet network characteristics for each news category. Number of nodes, edges, average degree, and degree heterogeneity of each network. The in- and out-degree heterogeneities are calculated by taking the average and standard error of 1000 independent samples of the degree heterogeneity ($\sigma(k_{in})/\langle k \rangle$ and $\sigma(k_{out})/\langle k \rangle$), each of which is computed on 78,911 samples with replacements from their respective degree distributions. 2016 data adapted from [21].

Username	News Media Category	2016	2020	Username	News Media Category	2016	2020
@foxandfriends	Right	10	NA	@nytpolitics	Left leaning	11	NA
@PalmerReport	Left	NA	23	@business	Center	8	NA
@OANN	Fake news	NA	8		Left leaning	NA	25
@ABCPolitics	Left leaning	9	52	@RawStory	Left	4	7
@USATODAY	Center	9	8	@gatewaypundit	Fake news	11	2
@FiveThirtyEight	Center	11	65	@PolitiFact	Left leaning	6	NA
@Mediaite	Left leaning	16	NA	@thehill	Center	2	1
@realDailyWire	Right	NA	13	@politico	Center	3	NA
@nytopinion	Left leaning	18	20		Left leaning	NA	10
@NYMag	Left	14	71	@dcexaminer	Right	3	10
@CREWcrew	Right leaning	NA	65	@newsmax	Extreme bias right	NA	6
	Center	NA	30	@FinancialTimes	Center	NA	18
	Left leaning	NA	12	@CNNPolitics	Center	4	NA
	Left	NA	21		Left leaning	NA	6
@BreitbartNews	Extreme bias right	3	2	@Reuters	Center	5	3
@Salon	Left	9	85	@NewDay	Center	25	NA
@Forbes	Right leaning	93	24	@TIME	Left	2	NA
@AP	Center	7	2	@VanityFair	Left	NA	20
@latimes	Left leaning	14	22	@ABC	Left leaning	3	4
@TheAtlantic	Left leaning	22	35	@HuffPost	Left	1	5
@theblaze	Right	21	33	@BuzzFeedNews	Left leaning	15	NA
@Rasmussen_Poll	Fake news	NA	14	@nypost	Right	5	NA
	Extreme bias right	NA	36		Right leaning	NA	1
	Right	NA	60	@mashable	Left	17	NA
	Right leaning	NA	20	@RT_America	Right leaning	5	NA
@NBCNews	Left leaning	4	8	@theintercept	Left	18	93
@CBSNews	Left leaning	7	9	@voxdotcom	Left leaning	8	NA
@NPR	Left leaning	27	7		Left	NA	11
@BuzzFeed	Left leaning	25	NA	@11thHour	Left	NA	24
@AP_Politics	Center	10	13	@HuffPostPol	Left	5	25
@Slate	Left leaning	5	NA	@wikileaks	Fake news	65	NA
	Left	NA	14		Extreme bias right	4	NA
@conserv_tribune	Fake news	21	NA		Right	16	NA
@NYDailyNews	Left leaning	13	NA		Center	21	NA
@foxnewspolitics	Right	23	NA		Left leaning	69	NA
@60Minutes	Left leaning	NA	24	@JudicialWatch	Fake news	NA	4
@FoxBusiness	Right	22	NA		Extreme bias right	53	NA
@Newsweek	Center	NA	6		Right	14	NA
	Left leaning	26	NA		Right leaning	20	NA
@thenation	Left	23	58	@NewYorker	Left	6	22
@guardian	Left leaning	12	95	@Telegraph	Right leaning	24	NA
@nytimes	Left leaning	1	2	@FoxNewsInsider	Right	6	NA
@RealClearNews	Right leaning	25	NA	@MSNBC	Left leaning	20	28
@bpolitics	Center	12	NA		Left	13	1
@businessinsider	Center	16	34	@washingtonpost	Left leaning	2	5
@WashTimes	Right leaning	2	6	@CNN	Center	1	NA
@SkyNews	Center	NA	16		Left leaning	NA	1
@TheEconomist	Center	NA	12	@DailyMail	Extreme bias right	NA	7
@APFactCheck	Center	NA	24		Right	7	NA
@BBCWorld	Center	24	23	@FoxNews	Right	1	5
@DailyCaller	Extreme bias right	2	NA	@thedailybeast	Left	3	2
	Right	NA	7	@RT_com	Fake news	NA	31
@thinkprogress	Left	10	NA		Right leaning	3	NA
@WSJopinion	Right leaning	12	67	@WSJPolitics	Right leaning	6	66
@TPM	Left	8	NA	@WSJ	Right leaning	1	2

Supplementary Table 5. News media categories and corresponding CI rankings for 87 influencers, for both the 2016 and 2020 U.S. Presidential elections. Influencers shown here are all established major news organizations and are verified on Twitter. Note that some influencers have more than one news category in which they are ranked, as they can influence multiple retweet networks. An entry is marked *NA* for a particular year and category if the influencer in question was not present as a top-100 influencer within that category during that year, but was within the top-100 during the other target year.

2016 → 2020	Fake & EB	Right	Right Leaning	Center	Left Leaning	Left	Inactive (2020)	Sum (2016)
Fake & EB	19846	17170	4002	1484	6046	526	159261	208335
Right	6758	10893	3353	1142	4950	366	112292	139754
Right leaning	803	705	994	663	3543	263	38810	45781
Center	2877	3946	3313	9425	63597	4337	417800	505295
Left Leaning	2382	3001	4140	12137	160241	10844	580205	772950
Left	546	543	1066	3568	46349	6540	198050	256662
Inactive (2016)	194478	244381	212214	291221	1722104	162182	/	2826580
Sum (2020)	227690	280639	229082	319640	2006830	185058	1506418	/

Supplementary Table 6. Shifts of users (absolute numbers) across news media categories from 2016 to 2020. Note that the label "Fake & EB" contains all the users from the fake news and the extremely biased left/right news categories. The inactive (2016) category indicates how many users who were non-existent or inactive in 2016 became active in their overlapping 2020 news media category. The inactive (2020) category indicates how many users active in their 2016 category became inactive in 2020.

Year	Modularity (SE)	Normalized Cut (SE)	Right Ratio	Left Ratio
2016	0.234 (0.004)	0.66 (0.03)	0.038	0.05
2020	0.236 (0.007)	0.58 (0.03)	0.038	0.08

Supplementary Table 7. Tabulated analysis of the similarity network using quotes instead of retweets for the top influencers. Note that the influencers here are determined by the CI rankings of the retweet networks. The similarity network is found for the 2016 and 2020 data. Using Louvain community detection reveals two communities with left- and center-oriented influencers in one community, and right- and fake-oriented influencers in the other. Left side of table: average modularity and average normalized cut, with the standard errors (SE) in parentheses, determined by taking sub-samples of influencers from the quote similarity network, detecting the two dichotomous communities with the sub-sampled quote similarity network, then recording their modularities and normalized cuts. Right side of table: ratio of quotes-to-retweets within the complete similarity network. Specifically, the number of user quotes of influencer tweets over the number of user retweets of influencer tweets. Right ratio indicates the average ratio for the community with right-oriented influencers. Left ratio indicates the average ratio for the community with left-oriented influencers. These ratios are found for both 2016 and 2020.

Year	Modularity	Normalized Cut
2016	0.373	0.253
2020	0.449	0.061

Supplementary Table 8. Modularity and normalized cut measures of the communities in the 2016 and 2020 networks in Fig. 5. These measures were run on the communities of both networks once, for all edges (both visible and hidden for sparsification). Consistent with the results of the main similarity networks, from 2016 to 2020, modularity increased while the normalized cut decreased.

A overall quotes/retweets

		2016	2020
from users	right	0.03	0.03
	left	0.05	0.04

B quotes/retweets

		2016		2020	
		to influencers			
		right	left	right	left
from users	right	0.02	0.19	0.02	0.49
	left	0.56	0.03	3.76	0.03

Supplementary Table 9. Comparison of fraction of retweets and quotes from users to influencers with different latent ideology estimates. Users and influencers are divided in two categories based on their ideology estimates, namely left (ideology <0) and right (ideology>0). Table **A** shows the overall proportion of quotes over retweets from users on the right and on the left revealing that the number of quotes represent only a small fraction ($\leq 5\%$) of the number of retweets. Table **B** shows the proportion of quotes over retweets from users to influencers for all pairs of ideology categories in 2016 and in 2020.

	user's distributions					influencers distributions				
	2016	95% CI	2020	95% CI	difference	2016	95% CI	2020	95% CI	difference
all	0.1086	[0.1082,0.1091]	0.1474	[0.1471,0.1477]	0.0388	0.1786	[0.1606,0.1965]	0.2091	[0.1907,0.2282	0.0305
common users	0.0941	[0.0934,0.0947]	0.1172	[0.1166,0.1178]	0.0231	0.1793	[0.1616,0.1979]	0.2143	[0.1952,0.2336]	0.0350
common influencers	0.1070	[0.1065,0.1076]	0.1830	[0.1825,0.1834]	0.0760	0.1641	[0.1290,0.1951]	0.1741	[0.1376,0.2122]	0.0100
common users and influencers	0.0947	[0.0940,0.0955]	0.1399	[0.1390,0.1406]	0.0452	0.1650	[0.1314,0.2034]	0.1719	[0.1379,0.2086]	0.0069

Supplementary Table 10. Hartigans' dip test statistics of the users and influencers latent ideology distributions. This analysis is done considering all users and influencers, only users that were present in 2016 and 2020, only influencers that were present in 2016 and 2020 and only users and influencers that were present in 2016 and 2020. 95% confidence intervals are computed from 1000 bootstrap samples with the bias-corrected and accelerated confidence intervals method.

References

[62] Webber, M., Moffat, A. & Zobel, J. A similarity measure for indefinite rankings. *ACM Trans. Inf. Syst.* **28**, 20 (2010).
